# Dynamic Additive Scanning for Precise Control in Electrospray Ionization Mass Spectrometry

**DOI:** 10.1021/acsmeasuresciau.5c00183

**Published:** 2026-02-04

**Authors:** I-Ting Wu, Decibel P. Elpa, Hsien-Ning Chien, Pawel L. Urban

**Affiliations:** Department of Chemistry, 150417 National Tsing Hua University 101, Section 2, Kuang-Fu Rd., Hsinchu 300044, Taiwan

**Keywords:** automation, charge state, electrospray ionization, mass spectrometry, optimization, protein, scan

## Abstract

In order to achieve optimum conditions of electrospray ionization (ESI) mass spectrometry (MS) methods, samples and mobile phases are often supplemented with acids, bases, supercharging reagents, and electrospray-friendly solvents. Typically, one pH or concentration of these additives is used in a given method, which is selected based on iterative optimization or literature. However, different pH values and concentrations of additives can be suitable for the analysis of different species, and subtle changes can bring different analytical information. Therefore, here we demonstrate a precise MS optimization system enabling dynamic scans of acid–base and additive concentrations in ESI-MS. In the case of low-molecular-weight analytes, MS signals can be enhanced by selecting the optimum conditions in single scans. The online acid–base scan showed enhancement factors of ∼5.4–7.9 for amino acids and related compounds, ∼44.7 for glutathione, and ∼4.5–10.3 for some tested phospholipids at 25%, 75%, and 90% base (stock solution volume ratio), respectively. For proteins, charge state distributions (CSDs) can be manipulated, bringing information on vulnerability of the protein tertiary structures to the changing environment. Multiple charging of cytochrome *c* and myoglobin was enhanced to varying degrees upon increasing concentrations of sulfolane and dimethyl sulfoxide, while increasing concentrations of organic solvents shifted CSDs to lower charge states. The setup for such measurements was constructed by using off-the-shelf components and by taking advantage of a simple Python code. Coupling online additive scans with ESI-MS streamlines optimization by eliminating the need for multiple sequential analyses of additives used to enhance signal intensity or induce supercharging.

## Graphical abstract

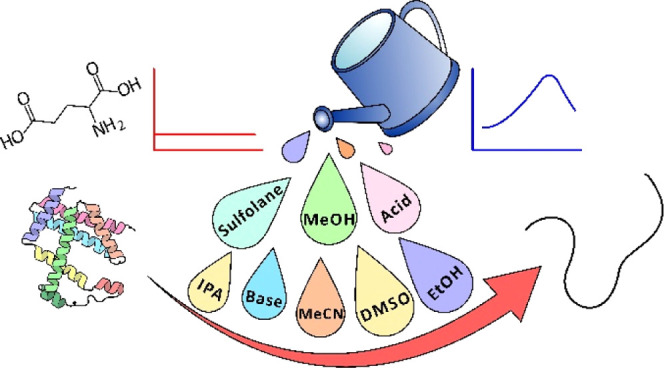

## Introduction

Electrospray ionization (ESI) mass spectrometry (MS) enables the analysis of a wide spectrum of molecular species—from small organic molecules to proteins—making it a central tool in modern analytical chemistry.^1–3^ Advances in chemical and biological research have driven the demand for sensitive ESI-MS detection of low-abundance analytes, such as single-cell metabolites, due to their critical roles in biological systems.^4,5^ A key factor that influences the sensitivity of ESI-MS is ionization efficiency (IE).^6^ IE indicates how effectively analyte molecules are transferred from the liquid phase to form gas-phase ions.^6,7^ Numerous parameters—including analyte properties, solvent composition, interface design, and flow rate, among others—affect IE.^3,6,7^ To achieve an optimal IE and enable accurate mass spectrometric analysis, it is a common practice to modify the samples and mobile phases with various additives, including acids, bases, and solvents. These additives significantly influence the response of the analyte due to their characteristics, concentration, and pH.^8–11^

The functional characteristics of proteins are determined by their three-dimensional structures.^12^ When the three-dimensional structure of a protein changes, its charge states also shift accordingly. ESI-MS is commonly used to study protein conformation by monitoring alterations in the charge state distributions (CSDs).^13^ In ESI-MS, the charge states of proteins can be significantly influenced by the sample matrix.^8,14^ It was reported in a previous study that the CSDs of myoglobin and cytochrome *c* shifted to lower charge states as the gas-phase basicity of the tested organic solvents increased.^8^ Moreover, adjusting the pH and concentration of additives in protein solutions influences their CSDs. For instance, when proteins are exposed to acids or bases—whether in solution or in vapor form—denaturation occurs, resulting in alterations to their conformations and CSDs.^15–19^ Another common approach for manipulating protein charge states is the addition of supercharging reagents.^20–24^ A key advantage of supercharging reagents is their ability to enhance the generation of multiply charged ions and improve signal intensity.^23^ There are several commonly used supercharging reagents, such as *m*-nitrobenzyl alcohol, dimethyl sulfoxide (DMSO), and tetramethylene sulfone (sulfolane).^21,23^ Notably, even when using the same supercharging reagent, the resulting CSDs vary significantly depending on the concentration used.^20,22^ These shifts not only reveal the dynamic structural changes of proteins under various conditions but also provide insights into how solution composition and protein conformation affect the ionization process in ESI-MS.^24–26^

Automation is becoming widely adopted in analytical chemistry workflows because it enhances efficiency, improves reproducibility, increases throughput, reduces reliance on specialized expertise, and minimizes human error.^27,28^ During the ESI-MS method development, multiple factors that affect IE and analyte detection are optimized. One effective approach—though time-consuming—is stepwise variable optimization, where one variable is adjusted one at a time to evaluate its specific effects. To save time and streamline this process, automated scanning systems are incorporated into the analytical workflows.^29,30^ For example, automated flow rate adjustment enables systematic characterization of how flow rate affects sensitivity and protein CSDs.^29,30^ An automated pH scan using a custom-built pH-meter enables precise and continuous modulation of pH to monitor reversibility in protein folding.^31^ Moreover, temporal concentration gradients can be created using simple fluidic systems coupled with MS.^32^

Here, we demonstrate a facile online system for conducting automated acid–base and additive concentration scans in ESI-MS. The system enables rapid optimization of ESI-MS methods in order to maximize MS signals. It also enables the alteration of CSDs of proteins. Unlike conventional approaches that require the preparation and infusion of multiple solutions, our system enables dynamic, automated adjustment of acid–base or additive levels within a single MS analysis. This eliminates the need for manual handling and analysis of multiple solutions, thereby streamlining the entire optimization process.

## Experimental Section

### Chemicals

Fluorescein (for fluorescence, free acid), Gly–His (GH, ≥ 98%), l-glutathione (GSH, reduced, ≥ 98%), l-lysine (≥98%, TLC grade), l-phenylalanine (98.5–101.0%), myoglobin (95–100%, from equine skeletal muscle, salt-free, lyophilized powder), and sulfolane (99%) were purchased from Sigma-Aldrich (St. Louis, MO, USA). DMSO (99.9%, spectroscopy grade), l­(+)-aspartic acid (≥98+%), l­(+)-glutamic acid (99%), l-histidine (98%), urocanic acid (98%), and ammonium acetate (≥98%, HPLC grade) were purchased from Acros Organics (Geel, Belgium). 1-Palmitoyl-2-oleoyl-*sn*-glycero-3-phosphate (16:0–18:1 PA; sodium salt), 1-palmitoyl-2-oleoyl-*sn*-glycero-3-phospho-l-serine (16:0–18:1 PS; sodium salt), 1,2-dioleoyl-*sn*-glycero-3-phospho-l-serine (18:1–18:1 PS; sodium salt), 1,2-dipalmitoyl-*sn*-glycero-3-phospho-(1′-*rac*-glycerol) (16:0–16:0 PG; sodium salt), 1,2-dioleoyl-*sn*-glycero-3-phosphoethanolamine (18:1–18:1 PE), and 1,2-dipalmitoyl-*sn*-glycero-3-phosphocholine (16:0–16:0 PC) were purchased from Avanti Polar Lipids (Alabaster, AL, USA). Cytochrome *c* (90%, from horse heart muscle) and formic acid (≥98%) were purchased from Thermo Scientific (Waltham, MA, USA). l-tyrosine (99%) was purchased from Alfa Aesar (Ward Hill, MA, USA). Methanol (MeOH, LC grade) and 2-propanol (IPA, LC–MS grade) were purchased from Merck (Darmstadt, Germany). Water (LC–MS grade) and acetonitrile (MeCN, LC–MS grade) were purchased from Fisher Chemical (Waltham, MA, USA). Ammonium hydroxide solution (30–33% NH_3_ in water) was purchased from Honeywell (Charlotte, NC, USA). Ethanol (EtOH, anhydrous, ≥ 99.5%) was purchased from Echo Chemical (Miaoli, Taiwan).

### Mass Spectrometry

MS analyses were performed by using a quadrupole time-of-flight (Q-TOF) mass spectrometer (LCMS-9030, Shimadzu Corporation, Kyoto, Japan) with an ESI source. The positive-ion mode was used with the ESI capillary voltage set to 4.0 kV. The nebulizing gas flow rate was 3.0 L min^–1^; the drying gas flow rate was 8.0 L min^–1^; and the heating gas flow rate was 8.0 L min^–1^. The desolvation line temperature was 250 °C, and the heat block temperature was 400 °C. The MS scan mode was used for all analyses. A scan range of mass-to-charge ratios (*m*/*z*) 20–1000 was used for amino acids and related compounds, peptides, and phospholipids; *m*/*z* 20–3000 for proteins. Data acquisition was conducted for 2 min with a MS sampling rate of 0.1 s.

### Online Additive Scanning System

A multichannel peristaltic pump (Reglo ICC Digital Pump, 4 channels, 12 rollers; Ismatec, Wertheim, Germany), a cross assembly (material: polyetheretherketone (PEEK); thru hole: 0.02 in with F-300 finger-tight fittings; part no. P-729; IDEX Health & Science, Rohnert Park, CA, USA), and a Q-TOF mass spectrometer were used to assemble the scanning system (Figure 1). The cross assembly (one port for sample, two ports for additives, and one port for sample-additive mixture) was fitted with three flared polyvinyl chloride Solva tubings (ID, 0.13 mm; OD, 1.80 mm; Pulse Instrumentation, Mequon, WI, USA) to inject sample and additive solutions and one PEEK tubing (length, 45 cm; ID, 0.13 mm; OD, 1.59 mm; GL Sciences, Tokyo, Japan) to deliver the sample-additive mixture from the cross-junction to the diverter valve of the ESI source. The end of each Solva tubing was connected to a section of polytetrafluoroethylene tubing (length 3 cm; ID 0.30 mm; OD 1.59 mm; Supelco, Merck, Darmstadt, Germany) by a fused silica capillary (length 4 cm; ID 0.15 mm; OD 0.375 mm; GL Sciences) to ensure a more stable connection at the cross-junction. The additive scanning system used an Arduino UNO R3 microcontroller board (Centenary Materials, Hsinchu, Taiwan) to precisely control the flow rate and trigger MS data acquisition (Figure S1). A Python script was used to initialize the serial communication with both the peristaltic pump and the microcontroller board and to trigger the microcontroller board to start MS data acquisition. The additive scan conditions are listed in Table S1.

**Figure 1:**
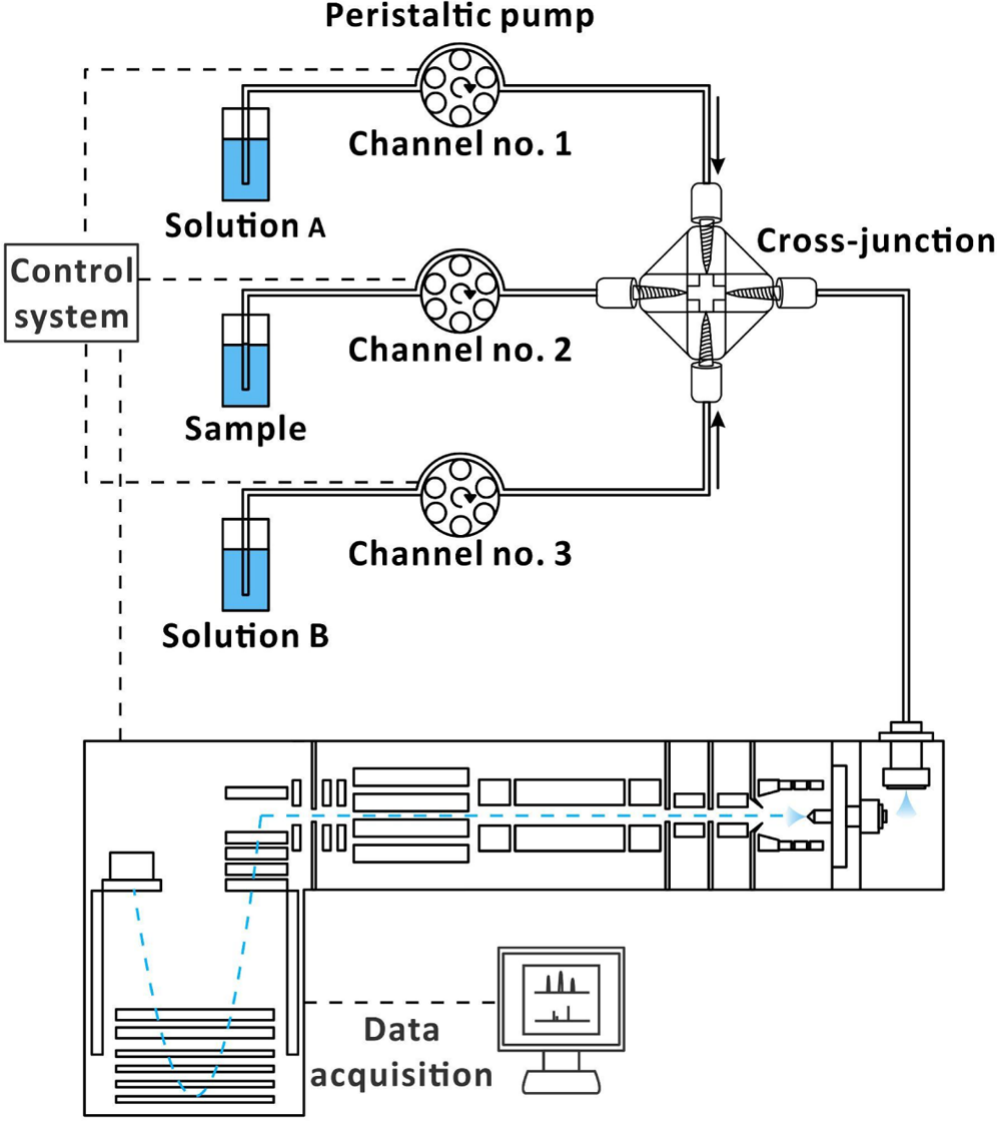
Scheme of the online additive scan setup for direct infusion MS analysis. The sample solution from the cross (sample mixed with additives) was delivered *via* a diverter valve to the ESI inlet.

A washing protocol was employed to flush the residual additives or analytes from the tubings. For acid–base scans and solvent concentration scans, channels 1–3 delivered 25% (v/v) aqueous MeOH for small-molecule analyses and 10% (v/v) aqueous MeOH for protein analyses. The tubings were washed for 5 min after four consecutive analyses. Supercharging reagents are more prone to residual retention in the tubings than acid–base additives. Accordingly, a more stringent washing protocol was implemented—after each analysis, the tubing was flushed for 30 s through channel 3 (20 μL min^–1^) only with 10% (v/v) MeOH, followed by a series of 5 min solvent washes after every four consecutive analyses. For supercharging reagent scans, channel 1 (reagent channel) was flushed with the corresponding solvents (10% (v/v) MeOH, 25% (v/v) IPA, water, and 10% (v/v) MeOH), while channels 2 and 3 were flushed with 10% (v/v) aqueous MeOH. For the 5 min washing protocol, the total flow rate for washing was maintained at 75 μL min^–1^ (channel 1: 20 μL min^–1^; channel 2: 35 μL min^–1^; and channel 3: 20 μL min^–1^).

### Data Treatment

During the 2 min data acquisition on the Q-TOF mass spectrometer, no sample was injected for the first 0.5 min. Step 1 of the scan was then initiated for 10 s, followed by sequentially varied steps (2–40) at 1 s intervals, with the final step lasting 20 s. Considering the system delay from the cross to the detector, step 1 effectively started at ∼0.78 min. Therefore, the data were analyzed from 0.93 to 1.61 min, covering the 41 s sequence (0–maximum% of additive). The extracted ion currents (EICs) of the analytes (mass extraction window of 10 ppm relative to the measured *m*/*z*) were exported from the mass spectrometer’s software (LabSolutions, version 5.113, Shimadzu Corporation, Kyoto, Japan) to ASCII files. Subsequently, the ASCII files were imported into OriginPro (2024b, OriginLab Corporation, Northampton, MA, USA) for data processing, plotting, and smoothing (Savitzky–Golay method; polynomial order, third; window, 35 points).

The enhancement factor (*EF*) for small molecules was calculated using the following formula

$$EF=\frac{\mathrm{I̅}}{\overset{®}{I_{0}\;}}$$ 1

where *I̅* is the mean signal intensity with additive, and $\overset{®}{I_{0}\;}$ is the mean signal intensity without additive. For each of the 41 experimental conditions (total duration: 41 s), 410 data points were recorded. The signal intensity for each condition was obtained by averaging 10 data points. For each condition, the final values were calculated as the mean across the three replicates.

For proteins, the weighted average charge state (*z*_av_) was calculated using the following equation

$$z_{av}=\frac{\Sigma \left(z_{i}I_{i}\right)}{\Sigma \left(I_{i}\right)}$$ 2

where *I*_*i*_ is the intensity of a peak for charge state *z*_*i*_ (*i* is the charge state number) at a given time point. Calculations of *z*_av_ were done using MATLAB (R2024b, MathWorks, Natick, MA, USA). For an example of the code, see the Supporting Information.

## Results and Discussion

### Online Acid–Base Scans for Analysis of Low-Molecular-Weight Compounds

A linear acid–base scan was performed to investigate how dynamic changes in the acid–base composition affect the IE and MS signal intensities of small molecules. We conducted a direct-infusion ESI-MS analysis with online acid–base ramp for amino acids and related compounds, peptides, and phospholipids (Figure 2). The automated stepwise introduction of 3 mM formic acid and 6 mM NH_3_(aq) into the sample resulted in analyte-specific changes in the signal intensity. For amino acids and related compounds, a 5 μM solution (aspartic acid, urocanic acid, lysine, glutamic acid, histidine, phenylalanine, and tyrosine in 25% (v/v) aqueous MeOH) was analyzed. The signal intensities of aspartic acid, urocanic acid, glutamic acid, phenylalanine, and tyrosine increased during the acid–base ramp compared to conditions without the ramp, where only the diluent (25% (v/v) aqueous MeOH) was used (Figures 2A and S2A,B). Aspartic acid and glutamic acid, which were undetectable without the ramp, showed detectable—though relatively low signals—under additive ramp conditions. The analytes exhibited their highest signal intensities at ∼25% base (stock solution volume ratio), suggesting that the acid–base scanning system enables automated determination of optimal signal enhancement conditions. The analytes exhibited varying degrees of enhancements (*EF*s from ∼5.4 to 7.9; Table S2). The combination of the analyte physicochemical properties such as molecular volume, hydrophobicity, surface activity, and protonation state dictates the varying degrees of enhancement or suppression observed during ESI-MS with acid–base scan.^33–37^ Low base percentage (or acidic condition) enhanced the ionization of phenylalanine and tyrosine by promoting protonation, complementing their surface affinities and, in the case of phenylalanine, its hydrophobicity.^33–35,37^ Acidic amino acids—such as aspartic acid and glutamic acid—were only detected under acid–base scan due to efficient protonation under low base conditions.^35^ Urocanic acid, which contains an imidazole group as a protonatable site, was similarly enhanced through stabilization of its protonated form.^35,38^ In contrast, lysine (*EF* ≈ 0.9) and histidine (*EF* ≈ 0.7) were suppressed under a low base percentage (Table S2). This may be attributed to the protonation of their basic side chains resulting in charge competition within ESI droplets, particularly in the presence of other surface-active analytes such as phenylalanine.^33–35,37^

**Figure 2:**
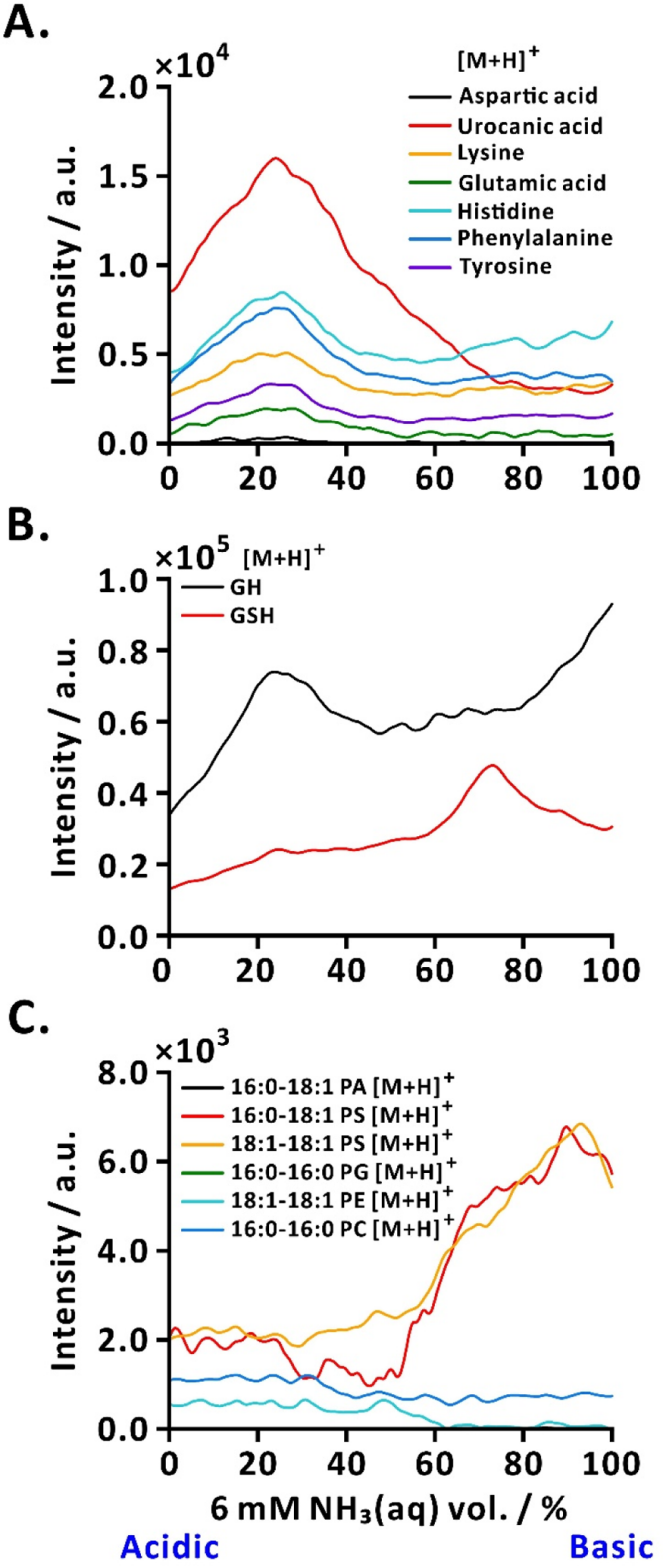
Online acid–base scans of small molecules. (A) EICs of amino acids and related compounds; (B) EICs of peptides; (C) EICs of protonated phospholipids. Sample: 5 μM aspartic acid, urocanic acid, lysine, glutamic acid, histidine, phenylalanine, and tyrosine in 25% (v/v) aqueous MeOH for amino acids and related compounds, 10 μM GH and GSH in 25% (v/v) aqueous MeOH for peptides, and 50 μM 16:0–18:1 PA, 16:0–18:1 PS, 18:1–18:1 PS, 16:0–16:0 PG, 18:1–18:1 PE, and 16:0–16:0 PC in 25% (v/v) aqueous MeOH for phospholipids. The NH_3_(aq) volume percentage, defined as the relative proportion of 6 mM NH_3_(aq) to 3 mM formic acid, was varied stepwise from 100% acid to 100% base over 41 s. One of three replicates is shown.

The online acid–base scan, coupled with ESI-MS, was also applied to peptides and phospholipids. Controlled addition of acid and base to a 10 μM solution of peptides (GH and GSH in 25% (v/v) aqueous MeOH) resulted in distinct signal responses between the two peptides (Figures 2B and S2C,D). While the signal intensity of GH was suppressed to varying degrees under dynamic acid–base ramp conditions, GSH exhibited increased signal intensity under the same conditions, with a maximum (*EF* ≈ 44.7) observed at ∼75% base (Table S2). We also analyzed a 50 μM solution of phospholipids (16:0–18:1 PA, 16:0–18:1 PS, 18:1–18:1 PS, 16:0–16:0 PG, 18:1–18:1 PE, and 16:0–16:0 PC in 25% (v/v) aqueous MeOH) using the dynamic acid–base ramp. Phospholipids generally exhibit optimal detection in either positive or negative mode ESI, demonstrating different ionization behaviors depending on their headgroup properties—whether zwitterionic or anionic.^39,40^ In this study, we focused on positive-mode ESI-MS, and we observed phospholipids that were not easily protonated but readily formed sodiated ions under dynamic acid–base ramp conditions (Figures 2C and S3).

In the absence of acid–base additives, protonated phospholipids were not detected (Figure S3A). During the acid–base ramp, protonated ions of 16:0–18:1 PS, 18:1–18:1 PS, 18:1–18:1 PE, and 16:0–16:0 PC were detected, while protonated ions of 16:0–18:1 PA and 16:0–16:0 PG remained undetected (Figures 2C and S3B). Overall, the EICs of both 16:0–18:1 PS [M + H]^+^ and 18:1–18:1 PS [M + H]^+^ exhibit a gradual increase in intensity up to ∼90% base, with the former displaying a slight decrease in intensity between ∼20% and 50% base (Figures 2C and S3B). The EIC of 18:1–18:1 PE [M + H]^+^ started to decrease at ∼50% base, while the EIC of 16:0–16:0 PC [M + H]^+^ showed no significant variation with the ramp (Figures 2C and S3B). Sodiated phospholipids were detected even without additives, except for 16:0–16:0 PG [M + Na]^+^ and 18:1–18:1 PE [M + Na]^+^ which were detected only under acid–base ramp conditions (Figure S3C,D). Notably, all sodiated phospholipids were detected during acid–base ramp, with signal enhancements (*EF*s from ∼4.5 to 10.3) for 16:0–18:1 PA [M + Na]^+^, 16:0–18:1 PS [M + Na]^+^, and 18:1–18:1 PS [M + Na]^+^ at ∼90% base, while no signal enhancement was observed for 16:0–16:0 PC [M + Na]^+^ at ∼90% base (Figure S3D and Table S2). These findings demonstrate that the dynamic acid–base ramp enables signal enhancement and serves as an effective automated scanning system for identifying optimal additive conditions for analytes—such as amino acids and related compounds, peptides, and phospholipids—with varying physicochemical properties, ionization preferences, and sensitivity to the acid–base composition. These findings also show the importance of accurately adjusting the additive concentration to achieve optimal analytical performance. This approach also has the potential to improve the analyte coverage by enhancing the detection of compounds that would otherwise be undetectable without additives. Our system relies on controlled delivery and mixing of solutions to generate efficient additive gradients. Accordingly, the flow system was evaluated for mixing efficiency, reproducibility, and carryover. See Supporting Information for evaluation details of the controlled flow system.

### Online Acid–Base Scans for Analysis of Proteins

We also conducted ESI-MS with an automated acid–base additive scan to investigate the CSDs of cytochrome *c* and myoglobin. A similar additive ramp as the one used for small molecules was employed to characterize protein CSD under varying acid–base conditions. Here, ammonium acetate was added as the background electrolyte to stabilize protein sample solutions.^41,42^ We compared 1 and 5 mM ammonium acetate to determine the optimum electrolyte concentration. The addition of 5 mM ammonium acetate stabilized the tested proteins (10 μM cytochrome *c* or myoglobin) during the acid–base ramp (Figure S4). Both cytochrome *c* and myoglobin gradually decreased their *z*_av_ from ∼9.5 for cytochrome *c* and ∼12.9 for myoglobin to ∼8.7 and ∼12.3, respectively, at ∼80–100% base, which is consistent with the values observed under additive-free conditions. In contrast, the CSDs of proteins varied with the acid–base ramp in the presence of 1 mM ammonium acetate (Figures 3 and S5). In this study, our goal was to investigate the influence of controlled addition of acid and base to protein CSD. Therefore, 1 mM ammonium acetate was selected as the electrolyte for representative online acid–base scans of proteins.

**Figure 3:**
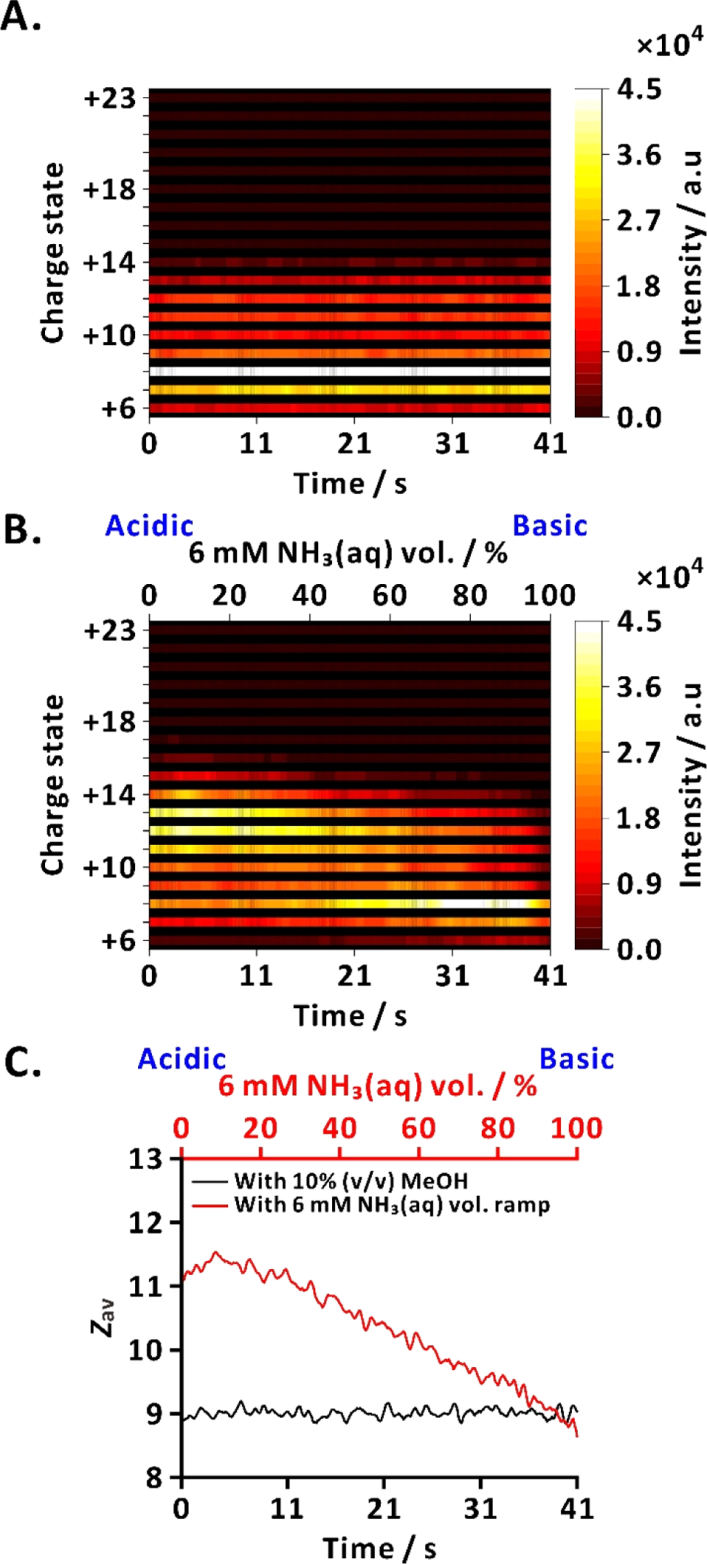
Comparison of cytochrome *c* direct infusion MS analyses without online acid–base scan (solvent: 10% (v/v) aqueous MeOH, constant flow rate) and with online acid–base scan. (A) Heatmap of charge state intensities (+6 to +23) without acid–base scan; (B) heatmap of charge state intensities (+6 to +23) under acid–base scan; (C) evolution of *z*_av_ under both conditions. Sample: 10 μM cytochrome *c* in 10% (v/v) aqueous MeOH with **1 mM ammonium acetate**. The NH_3_(aq) volume percentage, defined as the relative proportion of 6 mM NH_3_(aq) to 3 mM formic acid, was varied stepwise from 100% acid to 100% base over 41 s. The black lines and the thickness of the colored lines in (A,B) have no scientific meaning. One of three replicates is shown.

Without the acid–base ramp, cytochrome *c* remained partially unfolded (*z*_av_ ≈ 9.0) across the scan (Figure 3A,C). In all cases, both proteins were already partially unfolded, even without the additives, because of the heated ESI interface. With the acid–base ramp, higher charge states (+11 to +13) were dominant at low base levels (0–45%), corresponding to unfolded conformations of cytochrome *c* (Figure 3B,C; *z*_av_ ≈ 10.6–11.5). The *z*_av_ increased slightly from ∼11.1 to ∼11.5 during the initial phase of the ramp and then gradually decreased as the solution became more basic. At 45–100% base, +8 became the highest intensity charge state and *z*_av_ decreased to ∼8.7, indicating a CSD shift from unfolded to partially unfolded cytochrome *c* (Figure 3B,C).

A CSD shift was also observed for myoglobin under the acid–base ramp (Figure S5). At the beginning of the ramp, high-charge ions (centered around +15) dominated, corresponding to the unfolded state (*z*_av_ ≈ 13.5). As the base percentage increased, the distribution gradually shifted toward lower charge states. At ∼70–100% base, the highest intensity charge state shifted to +10, with *z*_av_ decreasing to ∼11.8, indicating a partially unfolded myoglobin. The heatmaps thus visualize the transition from unfolded to partially unfolded protein conformations across the ramp (Figures 3B and S5B). For myoglobin, the *z*_av_ initially increased from ∼13.5 to ∼14.5 during the early phase of the ramp, followed by a gradual *z*_av_ decrease as the solution became more basic (Figure S5C). This trend indicates a shift from unfolded to partially unfolded myoglobin under increasing base percentage of the acid–base ramp, consistent with the results observed for cytochrome *c*. Such observation is in line with the reported pH-induced protein conformational changes, where both myoglobin and cytochrome *c* undergo acid-induced unfolding at low pH, or partial refolding or stabilization at higher pH values.^16,19,31,43^ Although we did not directly measure solution pH, our system enabled monitoring of these conformational transitions in real time through *z*_av_ shifts during the online acid–base scan.

Additionally, we conducted acid–base scans on cytochrome *c* solution prepared under conditions representative of standard protein sample preparation, including higher ammonium acetate concentrations (20–100 mM, five levels) and the presence of acetonitrile (e.g., 30% v/v), which is typical of reversed-phase liquid chromatography (LC; Figures S6 and S7). With an acid–base ramp, the intensity of charge state +7 was consistently higher than +12 across 20–100 mM ammonium acetate (Figure S6). The EICs did not vary during the ramp. Consistent with our previous comparison between 1 and 5 mM ammonium acetate (Figures 3 and S4), higher ammonium acetate concentrations stabilized cytochrome *c*. This observation agrees with previous reports describing enhanced protein stability upon the addition of ammonium acetate.^41,42^ If the goal of an experiment is to observe changes in CSD during an additive scan, the protein sample can be diluted or subjected to dialysis prior to analysis by MS.

For the experiment on cytochrome *c* with 30% (v/v) MeCN, we used a 2× higher concentration of acid and base. It is common practice to add formic acid in LC mobile phases. Therefore, we added 0.1% (v/v) formic acid to the cytochrome *c* sample solution. With formic acid present in the sample, the base concentration in the base channel was increased to neutralize the added acid. The formic acid concentration in the acid channel was also increased to maintain equal acid and base concentrations and ensure consistency with the other acid–base ramp experiments. Notably, charge states +7 and +11 were detected with acid–base ramp (Figure S7) and exhibited increased signal intensities at higher base percentage (>40%). Signal fluctuations were observed for cytochrome *c* in the presence of 30% (v/v) MeCN, which may be attributed to bubble formation during mixing of the sample with the additive, clogging due to accumulation of residual analytes, and interactions between MeCN and the solvent delivery components (i.e., fused silica capillaries, Solva tubing, and PEEK tubing). Although such signal instability was present, it did not compromise the interpretation of the observed trends.

### Online Supercharging Reagent Scans for Analysis of Proteins

In addition to the acid–base scan, we conducted ESI-MS analyses using an automated supercharging reagent scan to investigate its effects on the CSD shifts of cytochrome *c* and myoglobin. Two supercharging reagents were introduced separately: 0–5% (v/v) sulfolane and 0–15% (v/v) DMSO. The addition of sulfolane did not enhance the signal intensities of either cytochrome *c* or myoglobin for charge states up to +15 and +24, respectively (Figures 4A,B and S8A,B). This may be attributed to the formation of protein–sulfate adducts during the ramp.^21,44^ However, for cytochrome *c*, some charge states exhibited their optimum intensities at a specific sulfolane concentration. This was particularly evident for charge states +17, + 18, + 19, and +20, with the highest intensities observed at ∼0.6% (v/v), ∼0.8% (v/v), ∼0.9% (v/v), and ∼1.0% (v/v) sulfolane, respectively (Figures 4B and S9). In addition, the intensities of charge states +21 to +23 increased during the sulfolane ramp compared to their intensities without sulfolane, indicating supercharging effects. The *z*_av_ increased from ∼15.9 to ∼18.0 at ∼1% (v/v) sulfolane. At concentrations above 1% (v/v), *z*_av_ remained constant, which suggests that a further increase in sulfolane did not enhance multiple charging (Figure 4C). The *z*_av_ at 0% (v/v) sulfolane was already slightly higher than that observed under additive-free conditions, which may be attributed to diffusion effects. For myoglobin, the intensities of charge states +25 to +32 increased during the sulfolane ramp compared to their intensities without sulfolane (Figure S8A,B). The *z*_av_ increased from ∼20.3 to ∼22.5 at ∼1% (v/v) sulfolane (Figure S8C), consistent with the supercharging effect observed for cytochrome *c*.

**Figure 4:**
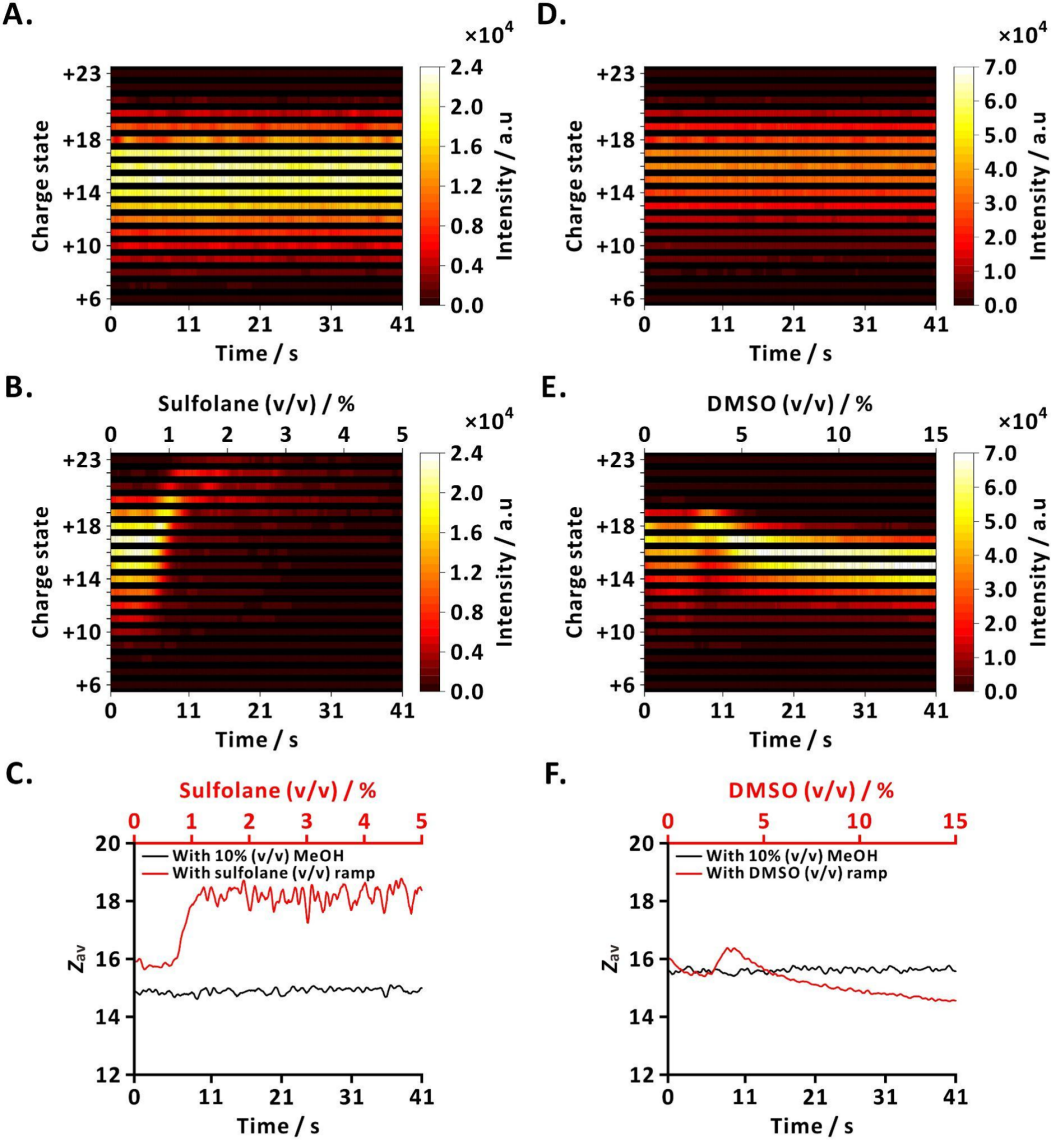
Comparison of cytochrome *c* direct infusion MS analyses without online supercharging reagent concentration scan (solvent: 10% (v/v) aqueous MeOH, constant flow rate) and with online supercharging reagent concentration scan. (A–C) Sulfolane concentration scan: (A) heatmap of charge state intensities (+6 to +23) without sulfolane concentration scan; (B) heatmap of charge state intensities (+6 to +23) under sulfolane concentration scan; (C) evolution of *z*_av_ under both conditions. (D–F) DMSO concentration scan: (D) heatmap of charge state intensities (+6 to +23) without DMSO concentration scan; (E) heatmap of charge state intensities (+6 to +23) under DMSO concentration scan; (F) evolution of *z*_av_ under both conditions. Sample: 10 μM cytochrome *c* with 0.1% (v/v) formic acid in 10% (v/v) aqueous MeOH. The supercharging reagent concentration was varied stepwise from 0% to the maximum level [sulfolane: 5% (v/v); DMSO: 15% (v/v)] over 41 s. The black lines and the thickness of the colored lines in (A,B,D,E) have no scientific meaning. One of three replicates is shown.

The addition of DMSO effectively increased the charge state signal intensities of the proteins. For cytochrome *c* (compared with the results obtained under constant 10% (v/v) MeOH; Figure 4D), signal intensities of some charge states were higher with varying DMSO concentrations (Figure 4E). At ∼3% (v/v) DMSO, some charge states (+18 and +19) reached their maximum intensities during the ramp, while other charge states (+13 to +17) reached their minimum. Moreover, above ∼3% (v/v) DMSO, the intensities of lower charge states (+13 to +15) kept increasing, resulting in a gradual shift in *z*_av_ from ∼16.4 at ∼3% (v/v) DMSO to ∼14.6 as DMSO percentage increased, indicating that higher concentrations of DMSO did not necessarily result in higher *z*_av_ (Figure 4F).

For myoglobin, the heatmaps revealed that the initial intensities of charge states were similar to the intensities without DMSO ramp, particularly below ∼3% (v/v) DMSO (Figure S8D,E). While the concentration of DMSO was above ∼3% (v/v), the intensities of higher charge states (+18 to +25) gradually decreased while the intensities of lower charge states (+13 to +17) gradually increased, with +17 as the highest intensity charge state at ∼10.5–15% (v/v) DMSO. Similar to cytochrome *c*, high concentrations of DMSO led to a decrease in *z*_av_, from an initial *z*_av_ of ∼19.4 to ∼16.2 at the end of the ramp (Figure S8F). The reduction in charge states—observed for both proteins—is consistent with previous reports showing that low levels of DMSO promote protein compaction, in contrast to the much higher concentrations (e.g., ≥30%) required to induce significant unfolding.^20,45^ Supercharging reagents—including the ones used in this study—are generally less volatile than water, causing them to concentrate in the evaporating ESI droplets and enhance protein charging.^20^ Sulfolane demonstrated more efficient supercharging than DMSO at comparable concentrations (Figures 4C,F and S8C,F), presumably due to its higher boiling point than DMSO (sulfolane, 287.6 °C at 760 Torr; DMSO, 189 °C at 760 Torr^20^). Previous studies have shown that the efficiency of supercharging positively correlates with the boiling points of these additives.^20,46^

### Online Solvent Scans for Analysis of Proteins

The effect of automatically varied concentrations of organic solvents on proteins (cytochrome *c* and myoglobin) in 10% (v/v) MeOH was evaluated (Table S1). Without an organic solvent ramp, cytochrome *c* in 10% (v/v) MeOH (Figure S10A) exhibited a stable *z*_av_ dominated by charge states from about +14 to +17. Under MeOH (6.36–25% (v/v), Figure S10B), EtOH (0–25% (v/v), Figure S10C), IPA (0–25% (v/v), Figure S10D), and MeCN (0–15% (v/v), Figure S10E) ramps, *z*_av_ stayed within a narrow range (∼14.5–16.0) across all solvents and conditions (Figure S10F). Among the tested solvents, small differences in intensities as the organic solvent concentration increased were observed, which may reflect solvent-dependent changes in the IE rather than conformational effects.

For myoglobin in 10% (v/v) MeOH (Figure S11A), the high intensity charge states were centered between +16 to +25. Under the MeOH ramp (6.36–25% (v/v), Figure S11B), *z*_av_ remained similar to that observed at constant 10% (v/v) MeOH. The minor differences were attributed to a decrease in charge state intensities with increasing MeOH concentration. When EtOH (0–25% (v/v), Figure S11C), IPA (0–25% (v/v), Figure S11D), and MeCN (0–15% (v/v), Figure S11E) were introduced, the intensities were notably lower than those observed with MeOH as the concentration of each organic solvent increased. Shifts in the maximum charge states and CSDs of cytochrome *c* and myoglobin to lower values upon exposure to vapors of organic solvents during ESI (or nanoESI) have been previously reported.^8,47,48^ Such observations were attributed to the gas-phase basicities of the solvents and their tendency to preferentially remove protons from ions in higher charge states,^8^ or to vapor-assisted slowing of desolvation, which stabilizes the proteins.^48^ Here, a similar trend was observed under an organic solvent ramp. While both constant 10% (v/v) MeOH and the MeOH ramp produced stable *z*_av_ values, EtOH, IPA, and MeCN ramps resulted in a modest decrease in *z*_av_ with increasing organic solvent concentration (Figure S11F). A similar effect was observed for cytochrome *c* (Figure S10F), although to a lesser extent than for myoglobin, primarily due to differences in structural stability and bonding.^49,50^ Cytochrome *c* exhibits higher structural stability than myoglobin because its heme is covalently attached through thioether linkages, while myoglobin binds heme noncovalently through weak hydrogen bonding and hydrophobic interactions, making it more susceptible to heme dissociation and unfolding under denaturing conditions.^51–53^ Moreover, the higher hydrophilicity of cytochrome *c* than myoglobin incurs higher solubility in solvents, potentially contributing to its stability.^50^

## Conclusions

We have demonstrated a straightforward and versatile approach for performing dynamic acid–base and additive concentration scans in ESI-MS. The system was assembled using widely available components and simple Python programming. Unlike earlier studies limited to a single additive concentration, the proposed method enables the controlled ramping of additive levels for gradual modulation of the solution environment. By automating this process, multiple conditions can be explored in a single ESI-MS analysis, thereby streamlining optimization. By taking advantage of temporal modifier ramps, this precision MS optimization approach eliminates the Edisonian trial-and-error style of method development. It facilitates the identification of optimal conditions for enhancing signals of low-molecular-weight analytes and provides insights into protein CSDs, enabling structural characterization under dynamic additive conditions. The online scanning system is particularly useful for analytes whose ionization efficiency is sensitive to solution conditions, such as pH, organic solvent content, and additive concentration. Practical applications include fast ESI-MS method optimization, protein charge-state probe and control, and matrix-effect screening. By continuously varying solvent strength or additive concentration during direct infusion ESI-MS, the development and suppression of ionization caused by salts, buffers, or coextracted components can be rapidly assessed, enabling the determination of optimized conditions in sample preparation, buffer selection, and additive use.

## Supplementary Material



## Notes

Additional results and discussion (evaluation of the controlled flow system); additive scan conditions; EFs for low-molecular-weight analytes; electronic circuit diagram; low-molecular-weight compound analyses with and without online acid–base scan; phospholipid analyses with and without online acid–base scan; proteins with 5 mM electrolyte analyzed with and without acid–base scan; myoglobin with 1 mM electrolyte analyzed with and without acid–base scan; cytochrome *c* with varying concentrations of ammonium acetate; cytochrome *c* with 30% (v/v) aqueous MeCN; myoglobin analyses with and without supercharging reagent concentration scan; charge state EICs of cytochrome *c*; cytochrome *c* analyses with and without solvent concentration scan; myoglobin analyses with and without solvent concentration scan; fluorescein fluorescence intensity images; EICs of three amino acids; online acid–base scan reproducibility; evaluation of analyte carryover after washing; repeatability of the online acid–base scan; and computer codes (PDF)
