# Supplementary material for: Dynamic Additive Scanning for Precise Control in Electrospray Ionization Mass Spectrometry
Source: ACS Meas Sci Au. 2026 Feb 4;6(2):445–53. doi: 10.1021/acsmeasuresciau.5c00183 (PMC13087944; doi:10.1021/acsmeasuresciau.5c00183)
Supplement: Supplementary file 1 [file tg5c00183_si_001.pdf]

SUPPORTING INFORMATION

Dynamic Additive Scanning for Precise Control in Electrospray Ionization Mass Spectrometry

I-Ting Wu, Decibel P. Elpa, Hsien-Ning Chien, Pawel L. Urban*

Department of Chemistry, National Tsing Hua University

101, Section 2, Kuang-Fu Rd., Hsinchu, 300044, Taiwan

* Corresponding author:

P.L. Urban (urban@mx.nthu.edu.tw)

ADDITIONAL RESULTS AND DISCUSSION

Evaluation of controlled flow system

To enable automated additive concentration gradients, a robust and reproducible flow system is required. We evaluated the additive scanning system for automated stepwise increase of additive solution and the subsequent mixing of sample, diluent, and additive solutions. The fluorescence images reveal a gradual and homogeneous increase in fluorescence intensity, indicating a corresponding increase in base percentage levels during the base ramp (**Figure S12**). Additionally, we have infused three standard analyte solutions *via* the channels used for additives and sample delivery (**Figure S13**). Individual standard solutions of histidine, phenylalanine, and tyrosine were delivered *via* channel 1 (flow rate increased from 0 to 20 μL min^{-1}), channel 2 (flow rate held constant at 35 μL min^{-1}), and channel 3 (flow rate decreased from 20 to 0 μL min^{-1}), respectively. As expected, phenylalanine showed a stable profile, histidine showed an ascending profile, while tyrosine showed a descending profile. Some deviations from linearity and fluctuations can be explained by the competition between the three species during the ESI process (suppression and enhancement).

The interday reproducibility of the additive scanning system was evaluated. The EICs of most amino acids and related compounds consistently reached optimum at ~ 20–30% base, demonstrating reproducibility of the online scanning system (**Figure S14**). Variations in signal intensity between days were observed, which were attributable to normal day-to-day fluctuations in MS instrument condition and performance. An increased signal of the analytes was observed after the washing step performed following four consecutive analyses with acid–base scan (**Figure S15A,B**). In contrast, comparable analyte signals were detected in the supercharging reagent scans before and after washing (**Figure S15C–F**). Notably, the observed carryover did not affect the repeatability of the acid–base scan (**Figure S16**). Extending the washing time and using different solvents may further reduce this effect. However, the overall analysis time must be considered.

ADDITIONAL TABLES

Table S1. Additive scan conditions and the corresponding diluent or solutions delivered *via* a multichannel peristaltic pump. The flow rates for channels 1, 2, and 3 were set as follows: channel 1 increased from 0 to 20 µL min^{-1}, channel 2 was held constant at 35 µL min^{-1}, and channel 3 decreased from 20 to 0 µL min^{-1}. Channel 2 contained the sample solution.

Scan	Additive concentration in the ramp	Channel 1	Channel 3
Acid–base	0 to 100% 6 mM NH_3(aq); relative proportion to 3 mM formic acid	6 mM NH_3(aq) in 25% or 10% (v/v) aqueous MeOH solution	3 mM formic acid in 25% or 10% (v/v) aqueous MeOH solution
Sulfolane concentration	0 to 5% (v/v) sulfolane	13.75% (v/v) sulfolane in 10% (v/v) aqueous MeOH solution	10% (v/v) aqueous MeOH solution
DMSO concentration	0 to 15% (v/v) DMSO	41.25% (v/v) DMSO in 10% (v/v) aqueous MeOH solution	10% (v/v) aqueous MeOH solution
MeOH concentration	6.36 to 25% (v/v) MeOH	51.25% (v/v) MeOH	water
EtOH concentration	0 to 25% (v/v) EtOH	68.75% (v/v) EtOH	water
IPA concentration	0 to 25% (v/v) IPA	68.75% (v/v) IPA	water
MeCN concentration	0 to 15% (v/v) MeCN	41.25% (v/v) MeCN	water

Table S2. *EF*s for amino acids and related compounds, peptides, and phospholipids. Some analytes—including aspartic acid, glutamic acid, sodiated forms of 16:0-16:0 PG and 18:1-18:1 PE, and all protonated phospholipids—were not detected without the acid–base ramp; therefore, their *EF*s were not calculated.

**Compound**	**Base**	***EF***
Aspartic acid	25%	undetectable without additives
Urocanic acid	25%	6.7
Lysine	25%	0.9
Glutamic acid	25%	undetectable without additives
Histidine	25%	0.7
Phenylalanine	25%	5.4
Tyrosine	25%	7.9
GH	75%	0.7
GSH	75%	44.7
16:0-18:1 PA $[M+Na]^+$	90%	4.5
16:0-18:1 PS $[M+Na]^+$	90%	10.3
18:1-18:1 PS $[M+Na]^+$	90%	6.6
16:0-16:0 PG $[M+Na]^+$	90%	undetectable without additives
18:1-18:1 PE $[M+Na]^+$	90%	undetectable without additives
16:0-16:0 PC $[M+Na]^+$	90%	1.0

ADDITIONAL FIGURES

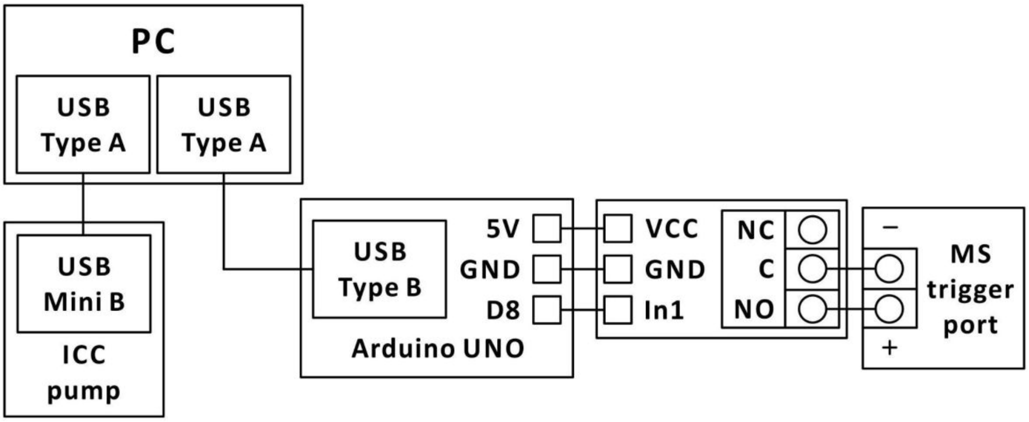

Figure S1. Electronic circuit diagram.

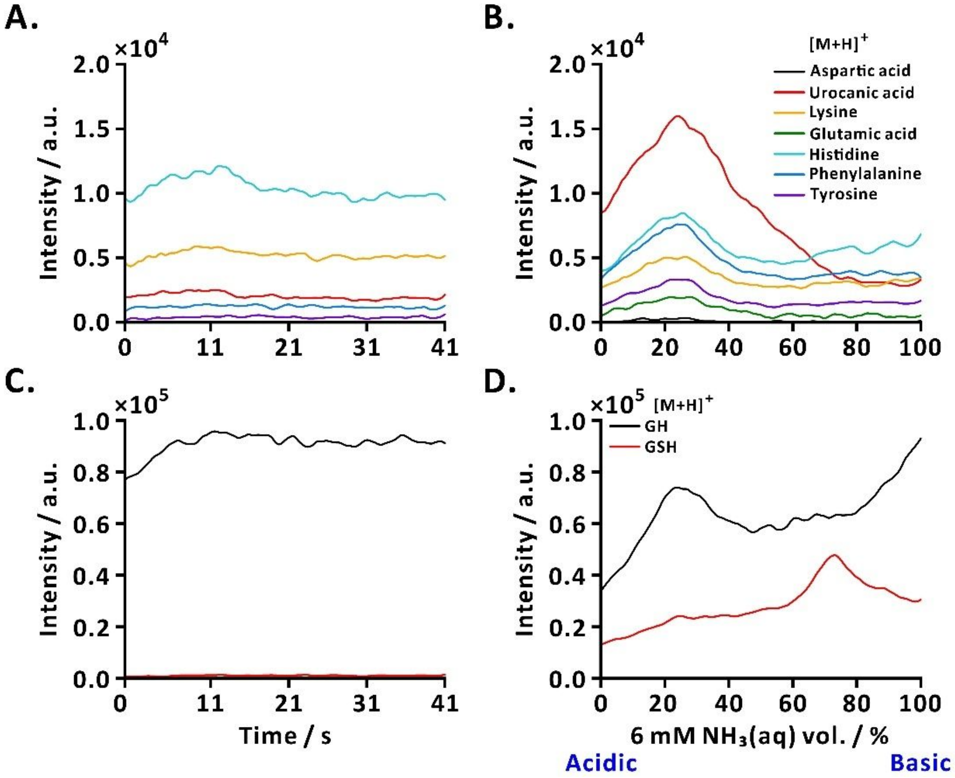

Figure S2. Comparison of low-molecular-weight compound direct infusion MS analyses without online acid–base scan (solvent: 25% (v/v) aqueous MeOH, constant flow rate) and with online acid–base scan. (A) EICs of amino acids and related compounds without acid–base scan; (B) EICs of amino acids and related compounds under acid–base scan; (C) EICs of peptides without acid–base scan; (D) EICs of peptides under acid–base scan. Samples: 5 µM aspartic acid, urocanic acid, lysine, glutamic acid, histidine, phenylalanine, and tyrosine in 25% (v/v) aqueous MeOH for amino acids and related compounds, and 10 µM GH and GSH in 25% (v/v) aqueous MeOH for peptides. The NH_3(aq) volume percentage, defined as the relative proportion of 6 mM NH_3(aq) to 3 mM formic acid, was varied stepwise from 100% acid to 100% base over 41 s. One of three replicates is shown. Note that (B) and (D) are the same results as **Figures 2A,B** and were included here for reference.

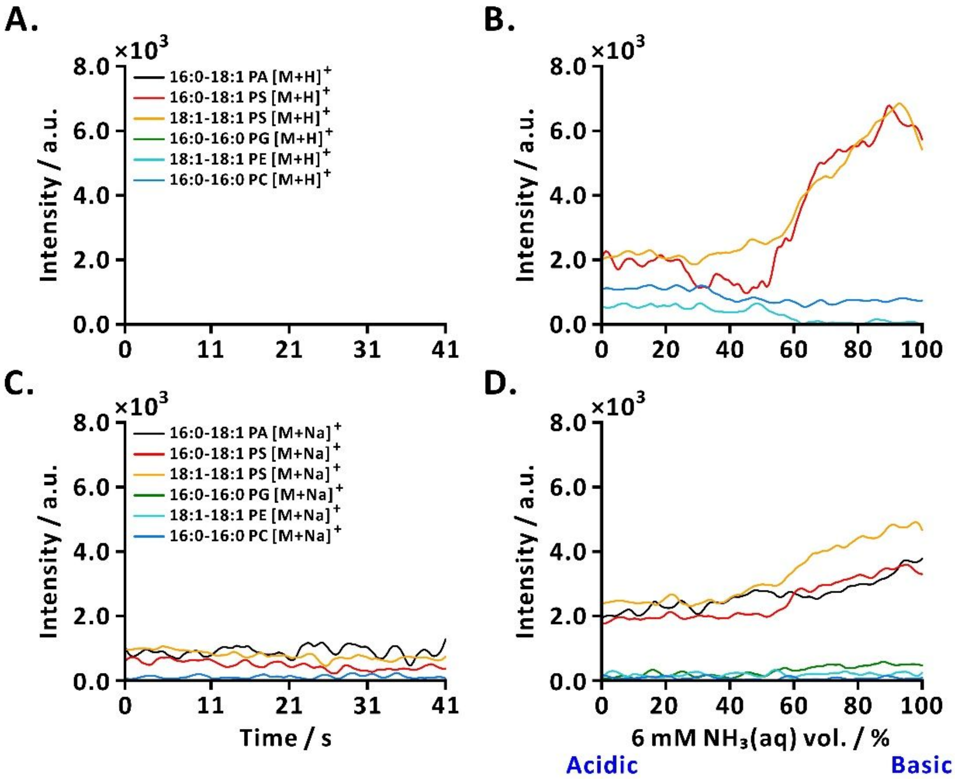

Figure S3. Comparison of phospholipids direct infusion MS analyses without online acid–base scan (solvent: 25% (v/v) aqueous MeOH, constant flow rate) and with online acid–base scan. (A) EICs of protonated phospholipids without acid–base scan; (B) EICs of protonated phospholipids under acid–base scan; (C) EICs of sodiated phospholipids without acid–base scan; (D) EICs of sodiated phospholipids under acid–base scan. Sample: 50 µM 16:0-18:1 PA, 16:0-18:1 PS, 18:1-18:1 PS, 16:0-16:0 PG, 18:1-18:1 PE, and 16:0-16:0 PC in 25% (v/v) aqueous MeOH. The NH_3(aq) volume percentage, defined as the relative proportion of 6 mM NH_3(aq) to 3 mM formic acid, was varied stepwise from 100% acid to 100% base over 41 s. One of three replicates is shown. Note that (B) is the same result as **Figure 2C** and was included here for reference.

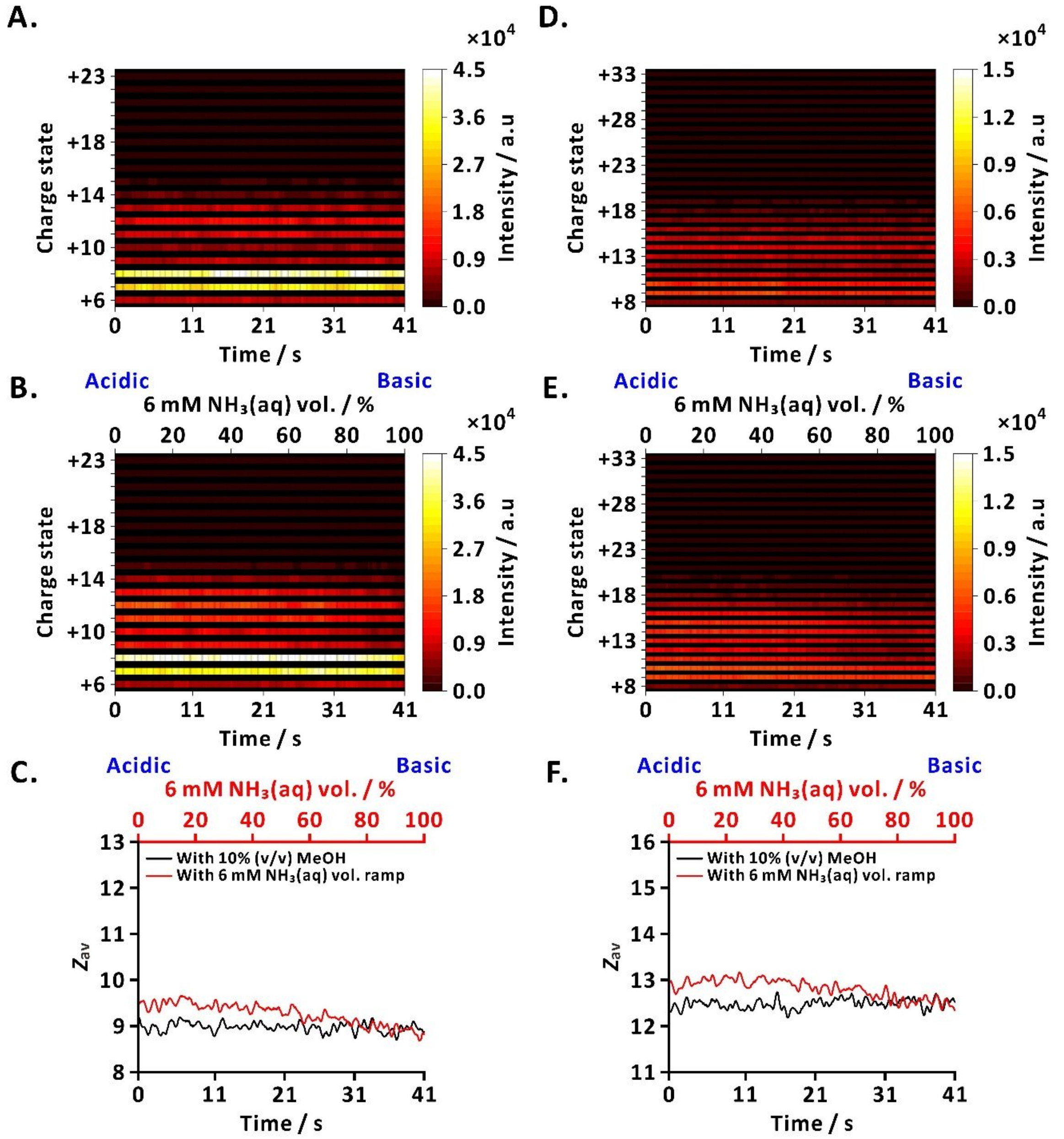

Figure S4. Comparison of cytochrome *c* and myoglobin direct infusion MS analyses without online acid–base scan (solvent: 10% (v/v) aqueous MeOH, constant flow rate) and with online acid–base scan. (A–C) Results for cytochrome *c*: (A) heatmap of charge state intensities (+6 to +23) without acid–base scan; (B) heatmap of charge state intensities (+6 to +23) under acid–base scan; (C) evolution of z_{av} under both conditions. (D–F) Results for myoglobin: (D) heatmap of charge state intensities (+8 to +33) without acid–base scan; (E) heatmap of charge state intensities (+8 to +33) under acid–base scan; (F) evolution of z_{av} under both conditions. Sample: 10 µM cytochrome *c* or myoglobin in 10% (v/v) aqueous MeOH with **5 mM ammonium acetate**. The NH_3(aq) volume percentage, defined as the relative proportion of 6 mM NH_3(aq) to 3 mM formic acid, was varied stepwise from 100% acid to 100% base over 41 s. The black lines and the thickness of the colored lines in (A,B,D,E) have no scientific meaning. One of three replicates is shown.

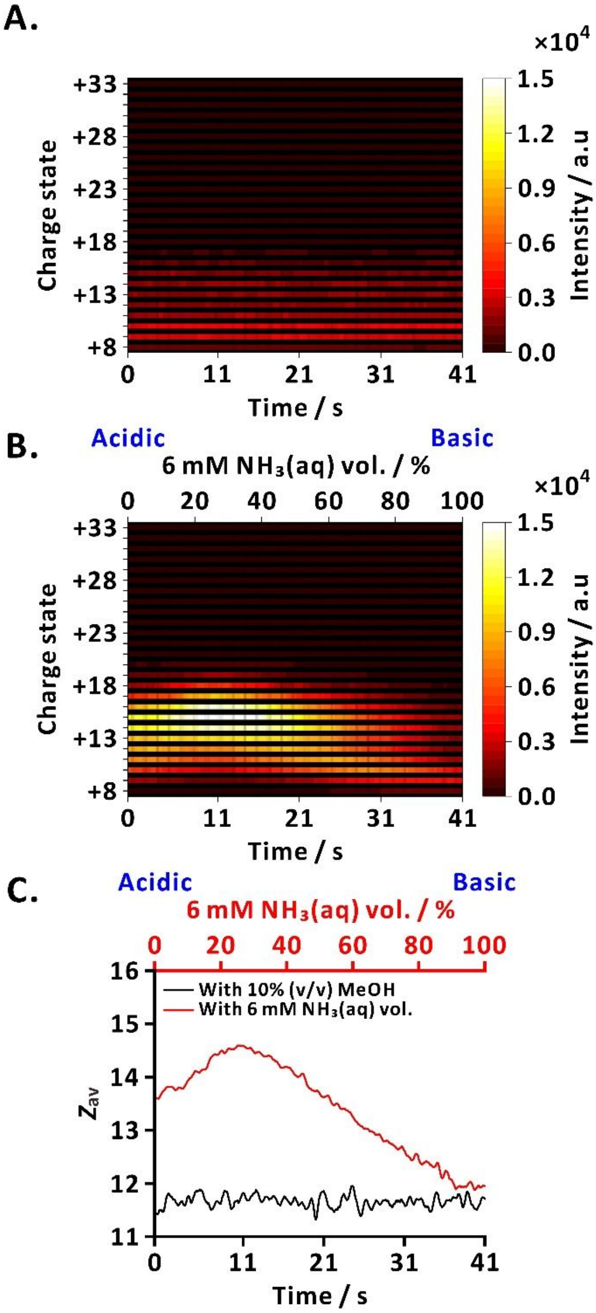

Figure S5. Comparison of myoglobin direct infusion MS analyses without online acid–base scan (solvent: 10% (v/v) aqueous MeOH, constant flow rate) and with online acid–base scan. (A) Heatmap of charge state intensities (+8 to +33) without acid–base scan; (B) heatmap of charge state intensities (+8 to +33) under acid–base scan; (C) evolution of z_{av} under both conditions. Sample: 10 μM myoglobin in 10% (v/v) aqueous MeOH with **1 mM ammonium acetate**. The NH_3(aq) volume percentage, defined as the relative proportion of 6 mM NH_3(aq) to 3 mM formic acid, was varied stepwise from 100% acid to 100% base over 41 s. The black lines and the thickness of the colored lines in (A,B) have no scientific meaning. One of three replicates is shown.

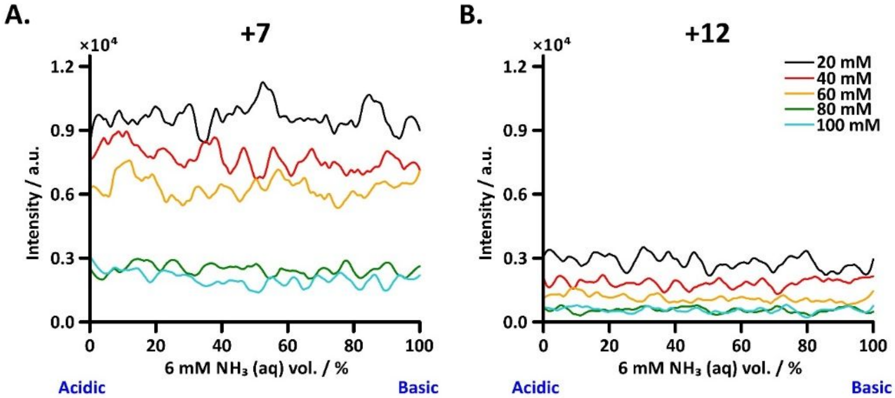

Figure S6. Direct infusion-MS using the online additive scanning system for the analysis of cytochrome *c* with varying concentrations of ammonium acetate. (A) EICs of charge state +7 and (B) EICs of charge state +12. Sample: 10 μM cytochrome *c* in 10% (v/v) aqueous MeOH with varying concentrations of ammonium acetate (20-100 mM, 5 levels). The NH_3(aq) volume percentage, defined as the relative proportion of 6 mM NH_3(aq) to 3 mM formic acid, was varied stepwise from 100% acid to 100% base over 41 s. One of three replicates is shown.

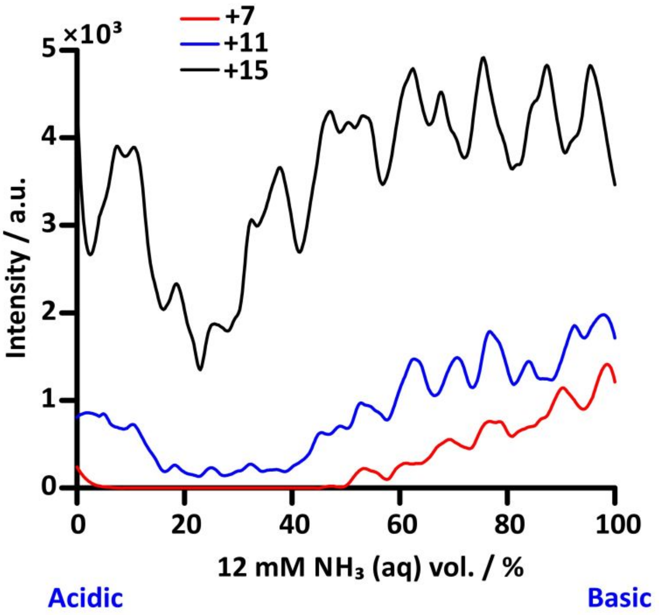

Figure S7. Representative charge state EICs of cytochrome *c* obtained by direct infusion-MS using the online additive scanning system. Sample: 10 μM cytochrome *c* with 0.1% (v/v) formic acid in 30% (v/v) aqueous MeCN. The NH_3(aq) volume percentage, defined as the relative proportion of 12 mM NH_3(aq) to 6 mM formic acid, was varied stepwise from 100% acid to 100% base over 41 s. One of three replicates is shown.

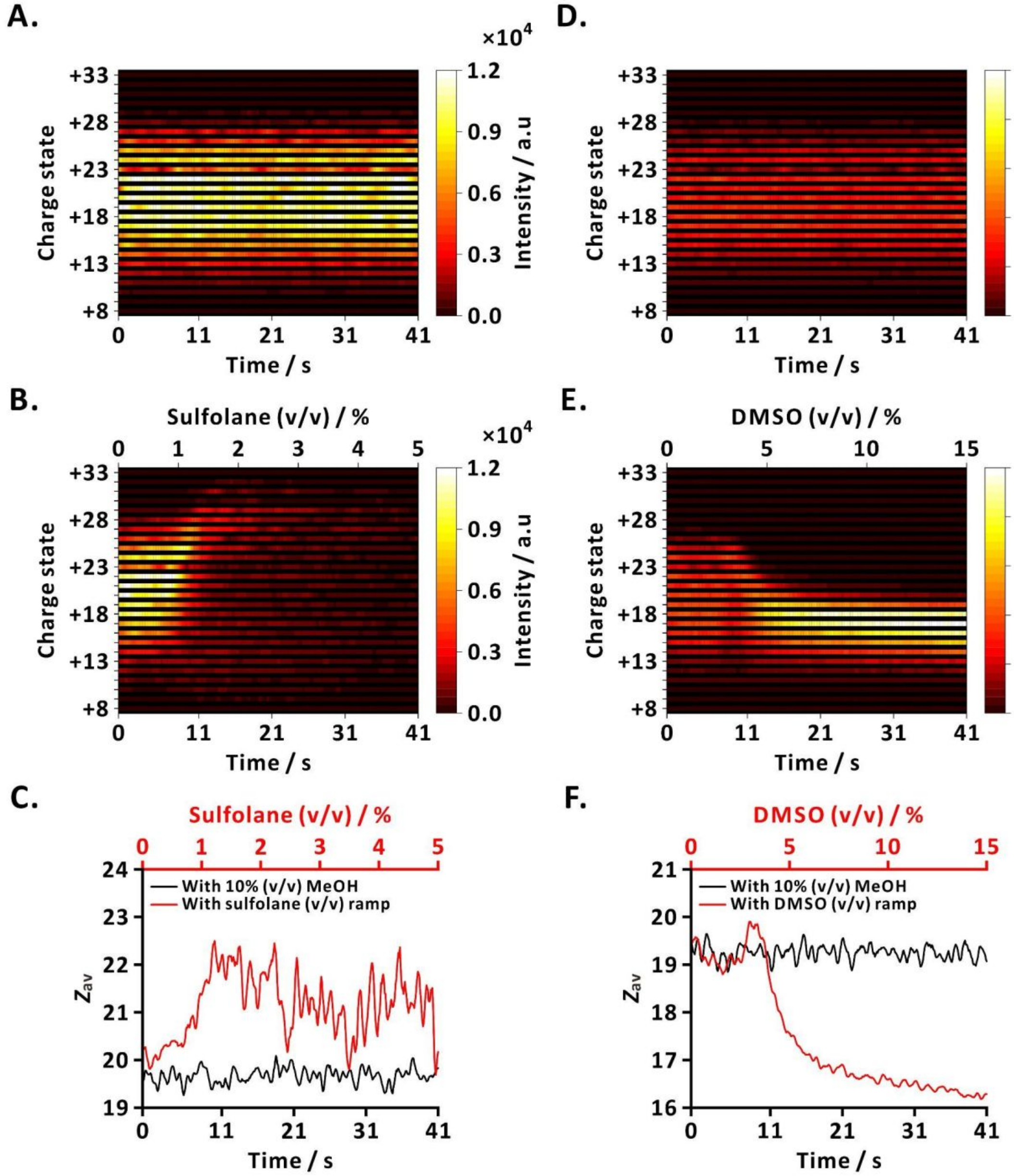

Figure S8. Comparison of myoglobin direct infusion MS analyses without online supercharging reagent concentration scan (solvent: 10% (v/v) aqueous MeOH, constant flow rate) and with online supercharging reagent concentration scan. (A–C) Sulfolane concentration scan: (A) heatmap of charge state intensities (+8 to +33) without sulfolane concentration scan; (B) heatmap of charge state intensities (+8 to +33) under sulfolane concentration scan; (C) evolution of z_{av} under both conditions. (D–F) DMSO concentration scan: (D) heatmap of charge state intensities (+8 to +33) without DMSO concentration scan; (E) heatmap of charge state intensities (+8 to +33) under DMSO concentration scan; (F) evolution of z_{av} under both conditions. Sample: 10 μM myoglobin with 0.1% (v/v) formic acid in 10% (v/v) aqueous MeOH. The supercharging reagent concentration was varied stepwise from 0% to the maximum level [sulfolane: 5% (v/v); DMSO: 15% (v/v)] over 41 s. The black lines and the thickness of the colored lines in (A,B,D,E) have no scientific meaning. One of three replicates is shown.

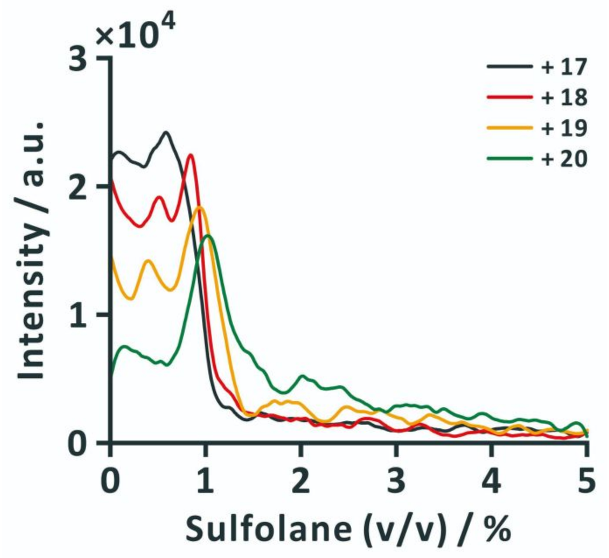

Figure S9. Representative EICs of cytochrome *c* which showed the changes of charge state intensities (+17 to +20) under online sulfolane concentration scan. One of three replicates is shown.

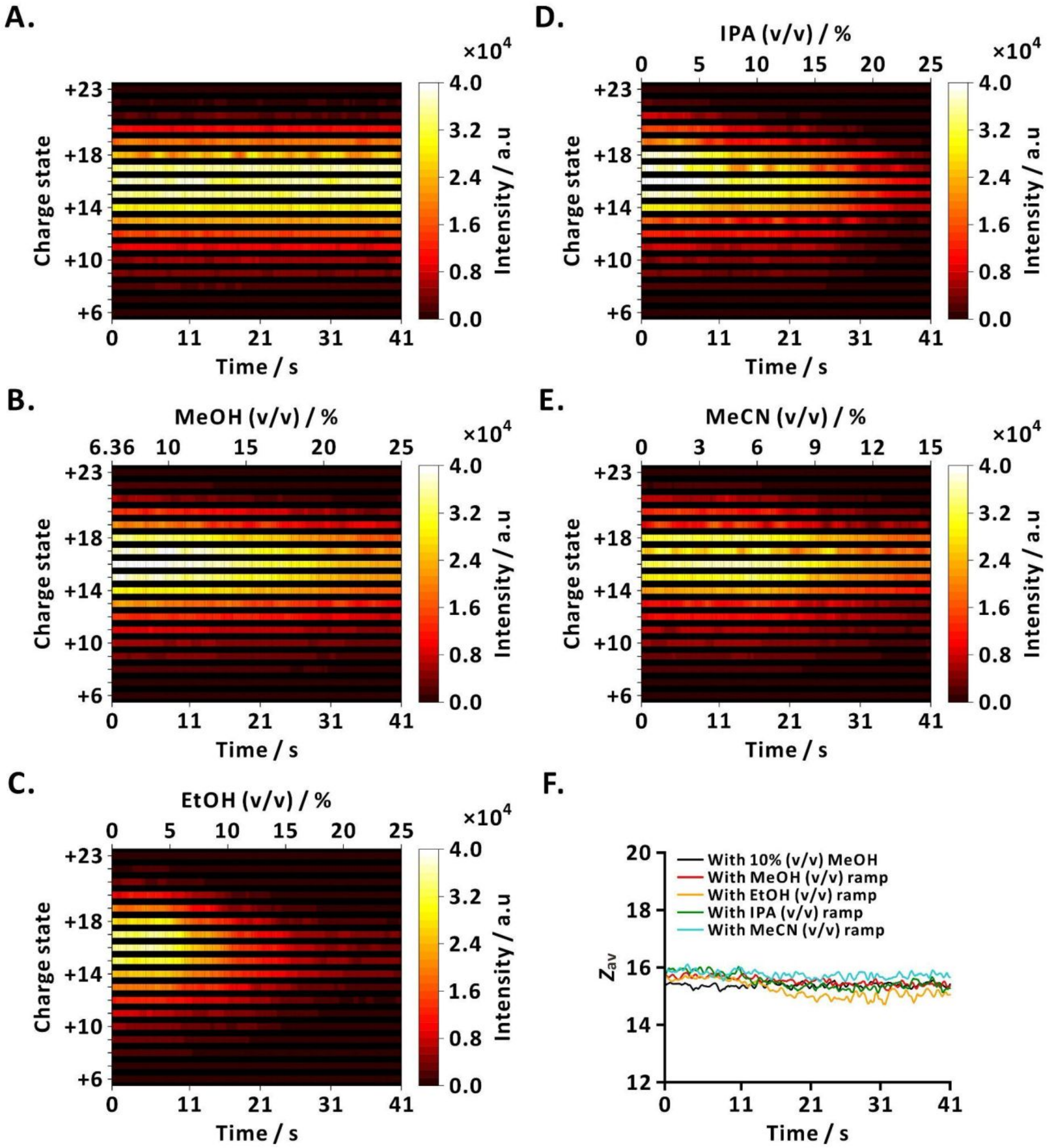

Figure S10. Comparison of cytochrome *c* direct infusion MS analyses without online solvent concentration scan (solvent: 10% (v/v) aqueous MeOH, constant flow rate) and with online solvent concentration scan. (A) Heatmap of charge state intensities (+6 to +23) without solvent concentration scan; (B) heatmap of charge state intensities (+6 to +23) under MeOH concentration scan; (C) heatmap of charge state intensities (+6 to +23) under EtOH concentration scan; (D) heatmap of charge state intensities (+6 to +23) under IPA concentration scan; (E) heatmap of charge state intensities (+6 to +23) under MeCN concentration scan; (F) evolution of z_{av} under the above conditions. Sample: 10 µM cytochrome *c* with 0.1% (v/v) formic acid in 10% (v/v) aqueous MeOH. The black lines and the thickness of the colored lines in (A–E) have no scientific meaning. One of three replicates is shown.

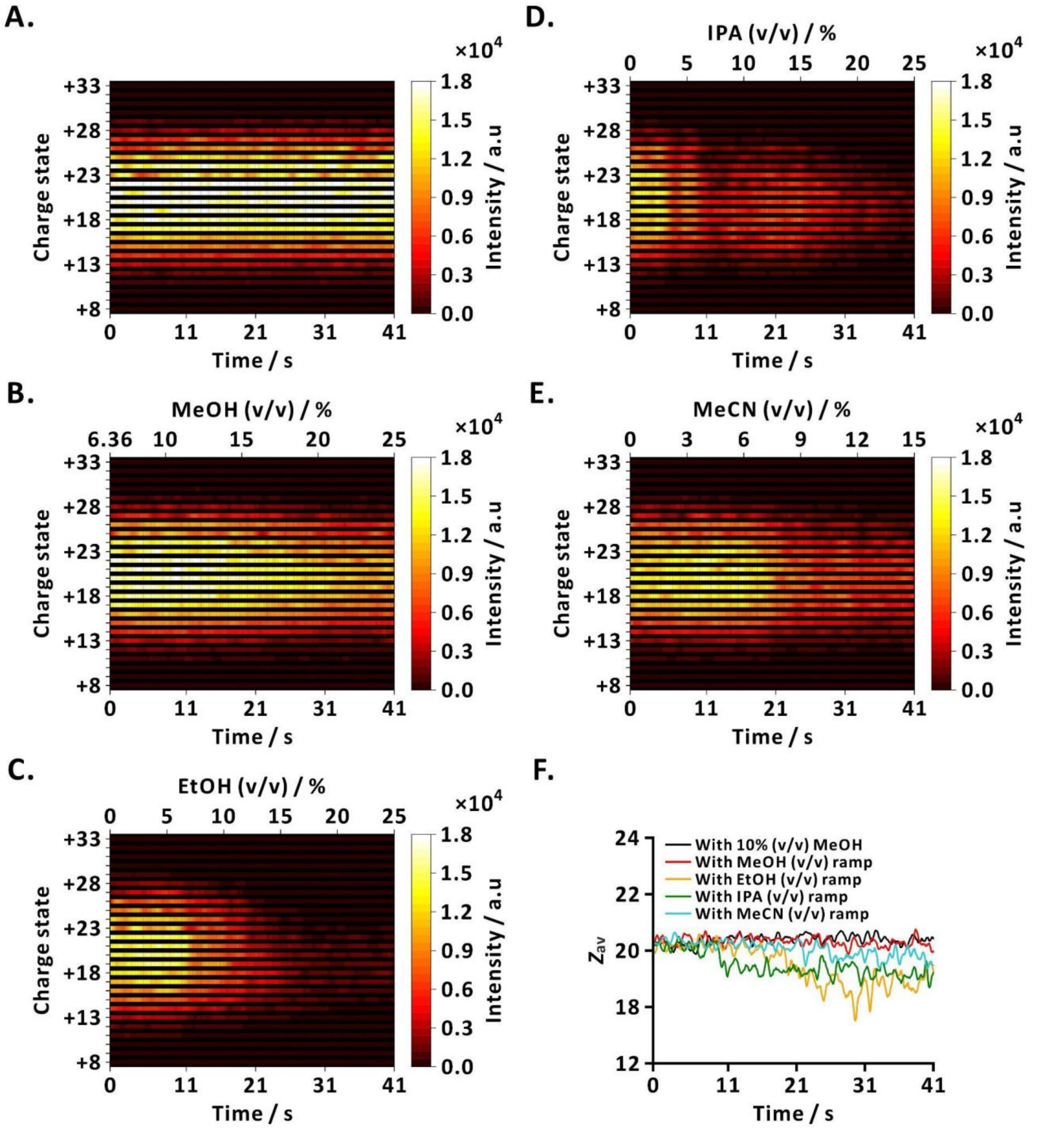

Figure S11. Comparison of myoglobin direct infusion MS analyses without online solvent concentration scan (solvent: 10% (v/v) aqueous MeOH, constant flow rate) and with online solvent concentration scan. (A) Heatmap of charge state intensities (+8 to +33) without solvent concentration scan; (B) heatmap of charge state intensities (+8 to +33) under MeOH concentration scan; (C) heatmap of charge state intensities (+8 to +33) under EtOH concentration scan; (D) heatmap of charge state intensities (+8 to +33) under IPA concentration scan; (E) heatmap of charge state intensities (+8 to +33) under MeCN concentration scan; (F) evolution of z_{av} under the above conditions. Sample: 10 μM myoglobin with 0.1% (v/v) formic acid in 10% (v/v) aqueous MeOH. The black lines and the thickness of the colored lines in (A–E) have no scientific meaning. One of three replicates is shown.

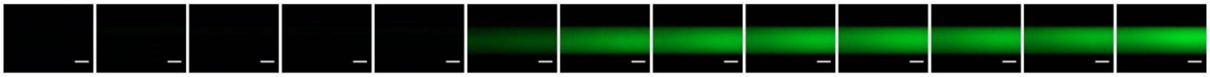

Figure S12. Verification of additive mixing in the proposed system by taking images of a capillary window in the downstream part of the manifold revealing changes in fluorescein fluorescence intensity. For these fluorometric measurements, a capillary window was placed within the optical path of a fluorescence microscope (Axio Imager M2; Carl Zeiss, Jena, Germany). The excitation wavelength was 460–500 nm, while the emission wavelength was 510–560 nm. Scale bars: 100 μm. The fused silica capillary (ID, 0.15 mm; OD, 0.375 mm) was connected to a cross (similar to the PEEK tubing described in the online additive scanning system section) to deliver the sample-additive mixture. The capillary was initially filled with 25% (v/v) aqueous MeOH. After 3 s when starting to snap photos, the liquid composition gradually changed *via* peristaltic pump. The peristaltic pump included 3 channels. Channel 1 was used to inject 6 mM NH_3(aq) in 25% (v/v) aqueous MeOH; channel 2 was used to inject 10 μM fluorescein in 25% (v/v) aqueous MeOH with 1 mM ammonium acetate; and channel 3 was used to inject 25% (v/v) aqueous MeOH. The flow rate for channel 1 was set from 0 to 20 μL min^{-1}; channel 2 was set to 35 μL min^{-1}; and channel 3 was set from 20 to 0 μL min^{-1}. The change was applied in a stepwise manner. Step 1 was initiated and maintained for 10 s, followed by steps 2 through 40, each applied at 1-s interval. The final step was held for 20 s. The exposure time was set to 200 ms. The time interval between snapshots was 5 s. Between trials, the tubing was washed following a protocol to prevent residual fluorescein from influencing the results. The total washing flow rate was set at 75 μL min^{-1} (channel 1: 20 μL min^{-1}; channel 2: 35 μL min^{-1}; channel 3: 20 μL min^{-1}). Each channel was sequentially flushed first with 3 mM formic acid, and then with 25% (v/v) aqueous MeOH for 5 min.

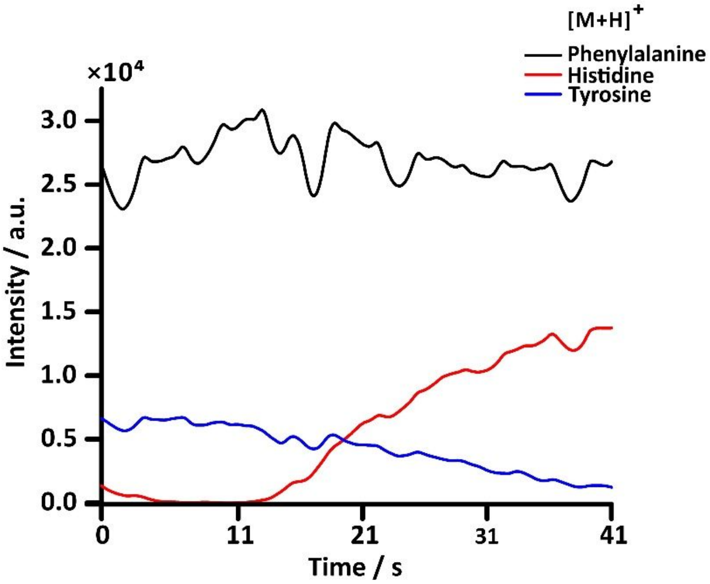

Figure S13. EICs of individual standard solutions of histidine, phenylalanine, and tyrosine (5 µM in 25% (v/v) aqueous MeOH with 0.1% (v/v) formic acid) obtained by direct infusion-MS using the online additive scanning system. Individual standard solutions of histidine, phenylalanine, and tyrosine were delivered *via* channel 1 (flow rate increased from 0 to 20 µL min^{-1}), channel 2 (flow rate held constant at 35 µL min^{-1}), and channel 3 (flow rate decreased from 20 to 0 µL min^{-1}), respectively. One of three replicates is shown.

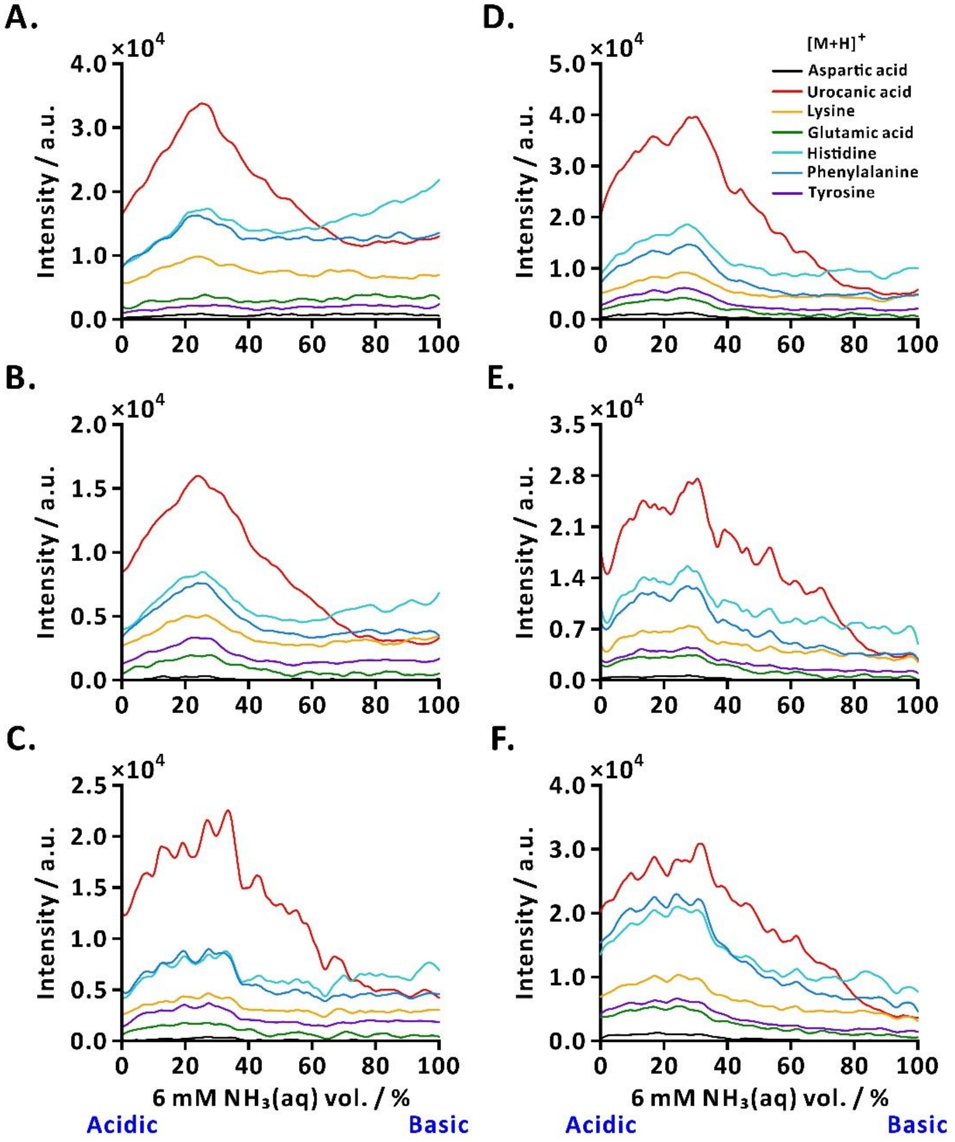

Figure S14. Representative results of low-molecular-weight compound analyses with online acid–base scan obtained on six different days. (A) January 16, 2025; (B) May 1, 2025; (C) May 2, 2025; (D) May 3, 2025; (E) May 14, 2025; (F) May 15, 2025. Sample: 5 µM aspartic acid, urocanic acid, lysine, glutamic acid, histidine, phenylalanine, and tyrosine in 25% (v/v) aqueous MeOH. The NH_3(aq) volume percentage, defined as the relative proportion of 6 mM NH_3(aq) to 3 mM formic acid, was varied stepwise from 100% acid to 100% base over 41 s. One of three replicates is shown.

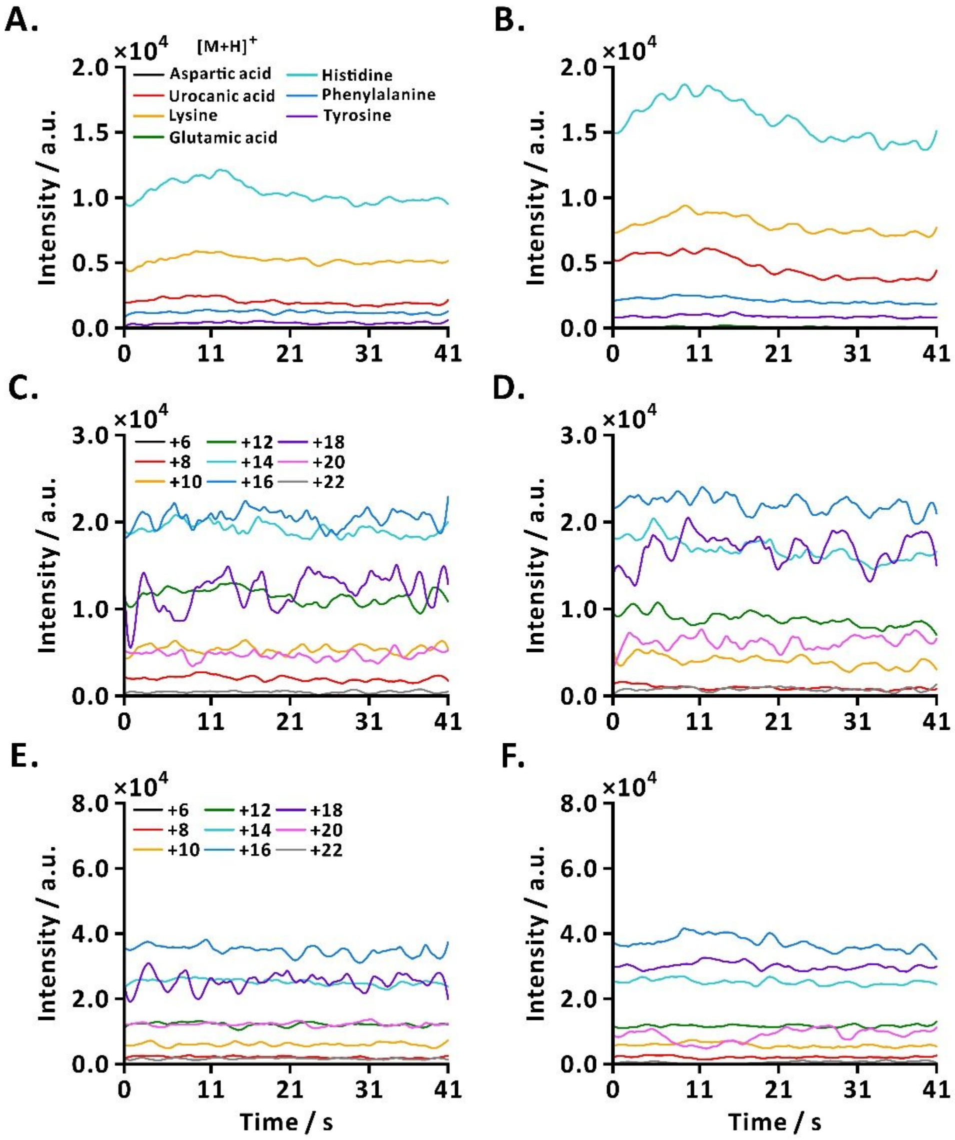

Figure S15. Signal intensities of samples without additive ramps, measured before (A, C, and E) and after (B, D, and F) additive use and subsequent washing. (A,B) Amino acids and related compounds before and after using formic acid and NH_3(aq); (C,D) cytochrome *c* before and after using sulfolane; (E,F) cytochrome *c* before and after using DMSO. The comparison evaluates the impact of additive residues retained in the tubing after washing. Samples: 5 μM aspartic acid, urocanic acid, lysine, glutamic acid, histidine, phenylalanine, and tyrosine in 25% (v/v) aqueous MeOH for amino acids and related compounds, and 10 μM cytochrome *c* with 0.1% (v/v) formic acid in 10% (v/v) aqueous MeOH. One of three replicates is shown.

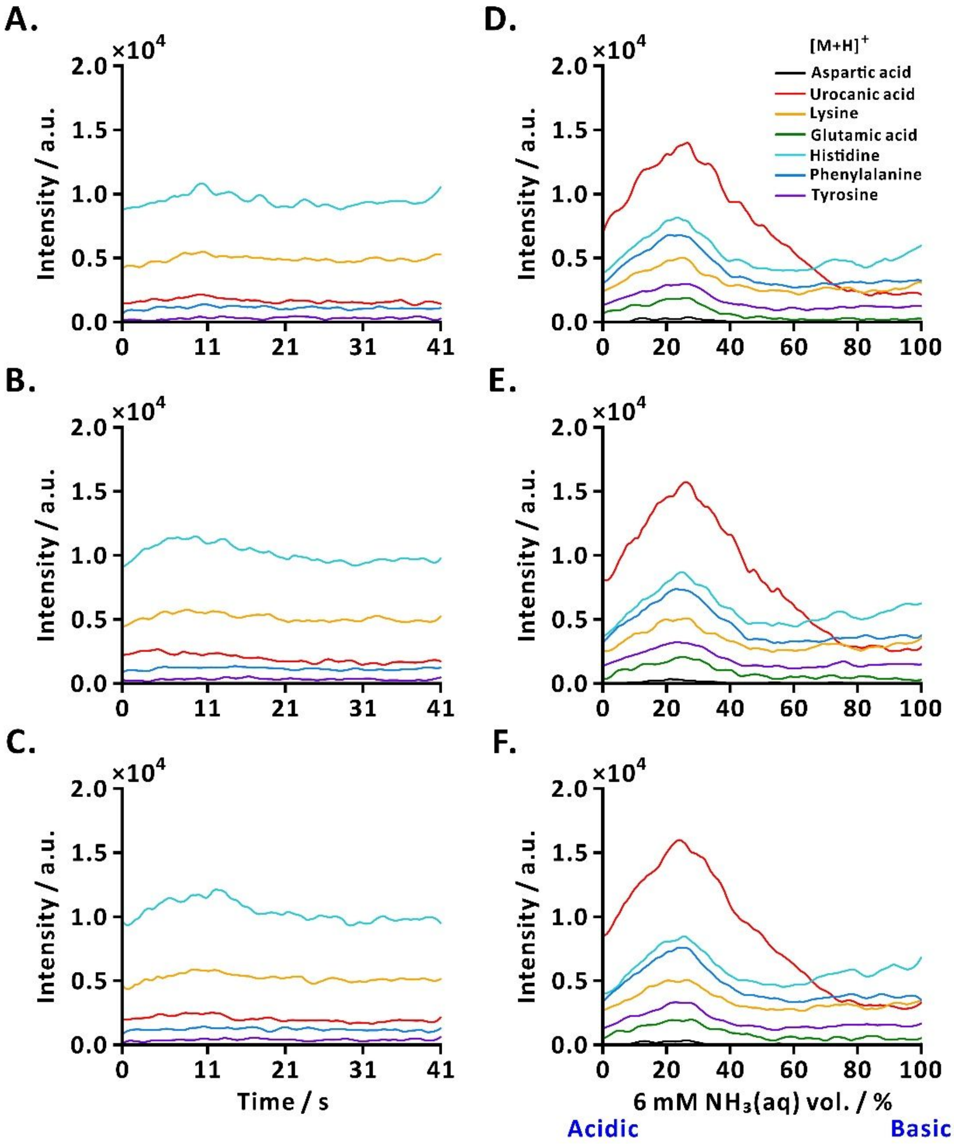

Figure S16. Three trials of results of low-molecular-weight compound analyses without online acid–base scan (solvent: 25% (v/v) aqueous MeOH, constant flow rate) and with online acid–base scan in one day. (A–C) Results without online acid–base scan: (A) trial 1; (B) trial 2; (C) trial 3. (D–F) Results with online acid–base scan: (D) trial 1; (E) trial 2; (F) trial 3. Sample: 5 µM aspartic acid, urocanic acid, lysine, glutamic acid, histidine, phenylalanine, and tyrosine in 25% (v/v) aqueous MeOH. The NH_3(aq) volume percentage, defined as the relative proportion of 6 mM NH_3(aq) to 3 mM formic acid, was varied stepwise from 100% acid to 100% base over 41 s.

COMPUTER CODES

Arduino code for the direct infusion MS analysis

```
const int QTOF = 8; // Assigning a pin to the MS data acquisition.
void setup() {
  Serial.begin(9600);
  pinMode(QTOF, OUTPUT);
  }
void loop() {
  if(Serial.available ()>0){
  int inByte = Serial.read();
  switch (inByte){

  case '1':
  digitalWrite(QTOF, 1);
  delay(500);
  digitalWrite(QTOF, 0);
  delay(500);
  break;
  }
}
}
```

Python code for the direct infusion MS analysis

```
import serial
import time

pump = serial.Serial("COM3", 9600, timeout=1)  # ICC pump
Arduino = serial.Serial("COM5", 9600, timeout=1)  # Arduino

def command(c):
    pump.write(b'%s\r\n' % (c))

def send_command(command):
    Arduino.write(command.encode())
    Arduino.flush()

time.sleep(2)
send_command(str(1))
time.sleep(30)
print('30 seconds')

command(b'2+0013')  # Set pump/channel 2 tubing ID: 0.13 mm
command(b'3+0013')  # Set pump/channel 3 tubing ID: 0.13 mm
command(b'4+0013')  # Set pump/channel 4 tubing ID: 0.13 mm
```

```
command(b'2J')  # Set rotation direction of pump/channel 2 to clockwise
command(b'3J')  # Set rotation direction of pump/channel 3 to clockwise
command(b'4J')  # Set rotation direction of pump/channel 4 to clockwise
command(b'2L')  # Set pump/channel 2 to RPM mode
command(b'3L')  # Set pump/channel 3 to RPM mode
command(b'4L')  # Set pump/channel 4 to RPM mode

command(b'3S003388')  # set RPM value 33.88 rpm for pump/channel 3
command(b'2S000000')  # set RPM value 00.00 rpm for pump/channel 2
command(b'4S002020')  # set RPM value 20.20 rpm for pump/channel 4
command(b'3H')  # start pumping (pump/channel 3)
command(b'2I')  # stop pumping (pump/channel 2)
command(b'4H')  # start pumping (pump/channel 4)
time.sleep(10)  # pumping with above parameters for 10 seconds

command(b'3S003388')
command(b'2S000018')
command(b'4S001969')
command(b'3H')
command(b'2H')
command(b'4H')
time.sleep(1)

command(b'3S003388')
command(b'2S000067')
command(b'4S001918')
command(b'3H')
command(b'2H')
command(b'4H')
time.sleep(1)

command(b'3S003388')
command(b'2S000117')
command(b'4S001866')
command(b'3H')
command(b'2H')
command(b'4H')
time.sleep(1)

command(b'3S003388')
command(b'2S000166')
command(b'4S001815')
command(b'3H')
command(b'2H')
command(b'4H')
time.sleep(1)

command(b'3S003388')
command(b'2S000215')
command(b'4S001764')
command(b'3H')
command(b'2H')
command(b'4H')
```

```
time.sleep(1)

command(b'3S003388')
command(b'2S000264')
command(b'4S001713')
command(b'3H')
command(b'2H')
command(b'4H')
time.sleep(1)

command(b'3S003388')
command(b'2S000314')
command(b'4S001662')
command(b'3H')
command(b'2H')
command(b'4H')
time.sleep(1)

command(b'3S003388')
command(b'2S000363')
command(b'4S001611')
command(b'3H')
command(b'2H')
command(b'4H')
time.sleep(1)

command(b'3S003388')
command(b'2S000412')
command(b'4S001559')
command(b'3H')
command(b'2H')
command(b'4H')
time.sleep(1)

command(b'3S003388')
command(b'2S000462')
command(b'4S001508')
command(b'3H')
command(b'2H')
command(b'4H')
time.sleep(1)

command(b'3S003388')
command(b'2S000511')
command(b'4S001457')
command(b'3H')
command(b'2H')
command(b'4H')
time.sleep(1)

command(b'3S003388')
command(b'2S000560')
command(b'4S001406')
```

```
command(b'3H')
command(b'2H')
command(b'4H')
time.sleep(1)

command(b'3S003388')
command(b'2S000609')
command(b'4S001355')
command(b'3H')
command(b'2H')
command(b'4H')
time.sleep(1)

command(b'3S003388')
command(b'2S000659')
command(b'4S001304')
command(b'3H')
command(b'2H')
command(b'4H')
time.sleep(1)

command(b'3S003388')
command(b'2S000708')
command(b'4S001253')
command(b'3H')
command(b'2H')
command(b'4H')
time.sleep(1)

command(b'3S003388')
command(b'2S000757')
command(b'4S001201')
command(b'3H')
command(b'2H')
command(b'4H')
time.sleep(1)

command(b'3S003388')
command(b'2S000807')
command(b'4S001150')
command(b'3H')
command(b'2H')
command(b'4H')
time.sleep(1)

command(b'3S003388')
command(b'2S000856')
command(b'4S001099')
command(b'3H')
command(b'2H')
command(b'4H')
time.sleep(1)
```

```
command(b'3S003388')
command(b'2S000905')
command(b'4S001048')
command(b'3H')
command(b'2H')
command(b'4H')
time.sleep(1)

command(b'3S003388')
command(b'2S000954')
command(b'4S000997')
command(b'3H')
command(b'2H')
command(b'4H')
time.sleep(1)

command(b'3S003388')
command(b'2S001004')
command(b'4S000946')
command(b'3H')
command(b'2H')
command(b'4H')
time.sleep(1)

command(b'3S003388')
command(b'2S001053')
command(b'4S000895')
command(b'3H')
command(b'2H')
command(b'4H')
time.sleep(1)

command(b'3S003388')
command(b'2S001102')
command(b'4S000843')
command(b'3H')
command(b'2H')
command(b'4H')
time.sleep(1)

command(b'3S003388')
command(b'2S001151')
command(b'4S000792')
command(b'3H')
command(b'2H')
command(b'4H')
time.sleep(1)

command(b'3S003388')
command(b'2S001201')
command(b'4S000741')
command(b'3H')
command(b'2H')
```

```
command(b'4H')
time.sleep(1)

command(b'3S003388')
command(b'2S001250')
command(b'4S000690')
command(b'3H')
command(b'2H')
command(b'4H')
time.sleep(1)

command(b'3S003388')
command(b'2S001299')
command(b'4S000639')
command(b'3H')
command(b'2H')
command(b'4H')
time.sleep(1)

command(b'3S003388')
command(b'2S001349')
command(b'4S000588')
command(b'3H')
command(b'2H')
command(b'4H')
time.sleep(1)

command(b'3S003388')
command(b'2S001398')
command(b'4S000536')
command(b'3H')
command(b'2H')
command(b'4H')
time.sleep(1)

command(b'3S003388')
command(b'2S001447')
command(b'4S000485')
command(b'3H')
command(b'2H')
command(b'4H')
time.sleep(1)

command(b'3S003388')
command(b'2S001496')
command(b'4S000434')
command(b'3H')
command(b'2H')
command(b'4H')
time.sleep(1)

command(b'3S003388')
command(b'2S001546')
```

```
command(b'4S000383')
command(b'3H')
command(b'2H')
command(b'4H')
time.sleep(1)

command(b'3S003388')
command(b'2S001595')
command(b'4S000332')
command(b'3H')
command(b'2H')
command(b'4H')
time.sleep(1)

command(b'3S003388')
command(b'2S001644')
command(b'4S000281')
command(b'3H')
command(b'2H')
command(b'4H')
time.sleep(1)

command(b'3S003388')
command(b'2S001693')
command(b'4S000230')
command(b'3H')
command(b'2H')
command(b'4H')
time.sleep(1)

command(b'3S003388')
command(b'2S001743')
command(b'4S000178')
command(b'3H')
command(b'2H')
command(b'4H')
time.sleep(1)

command(b'3S003388')
command(b'2S001792')
command(b'4S000127')
command(b'3H')
command(b'2H')
command(b'4H')
time.sleep(1)

command(b'3S003388')
command(b'2S001841')
command(b'4S000076')
command(b'3H')
command(b'2H')
command(b'4H')
time.sleep(1)
```

```
command(b'3S003388')
command(b'2S001891')
command(b'4S000025')
command(b'3H')
command(b'2H')
command(b'4H')
time.sleep(1)

command(b'3S003388')
command(b'2S001940')
command(b'4S000000')
command(b'3H')
command(b'2H')
command(b'4I')
time.sleep(20)

command(b'3S000000')
command(b'2S000000')
command(b'4S000000')
command(b'3I')
command(b'2I')
command(b'4I')
time.sleep(10)

print('finish')
```

Matlab code for calculating the average charge states of proteins

```
clear
clc

path = {
"D:\Lab_data\reprocessbysoftware\
Cytochrome c\20250109\pos-1mM_sample+solvent-3.lcd.txt";
};

opts = delimitedTextImportOptions("NumVariables", 3);
opts.DataLines = [11, Inf];
opts.Delimiter = "\t";
opts.VariableNames = ["Header", "VarName2", "VarName3"];
opts.VariableTypes = ["double", "double", "double"];
opts.ExtraColumnsRule = "ignore";
opts.EmptyLineRule = "read";
opts.ImportErrorRule = "omitrow";
opts.MissingRule = "omitrow";

x = 1200;

ranges = [
```

557, 557;
558, 558;
559, 559;
560, 560;
561, 561;
562, 562;
563, 563;
564, 564;
565, 565;
566, 566;
567, 567;
568, 568;
569, 569;
570, 570;
571, 571;
572, 572;
573, 573;
574, 574;
575, 575;
576, 576;
577, 577;
578, 578;
579, 579;
580, 580;
581, 581;
582, 582;
583, 583;
584, 584;
585, 585;
586, 586;
587, 587;
588, 588;
589, 589;
590, 590;
591, 591;
592, 592;
593, 593;
594, 594;
595, 595;
596, 596;
597, 597;
598, 598;
599, 599;
600, 600;
601, 601;
602, 602;
603, 603;
604, 604;
605, 605;
606, 606;
607, 607;
608, 608;
609, 609;

```
610, 610;
611, 611;
612, 612;
613, 613;
614, 614;
615, 615;
616, 616;
617, 617;
618, 618;
619, 619;
620, 620;
621, 621;
622, 622;
623, 623;
624, 624;
625, 625;
626, 626;
627, 627;
628, 628;
629, 629;
630, 630;
631, 631;
632, 632;
633, 633;
634, 634;
635, 635;
636, 636;
637, 637;
638, 638;
639, 639;
640, 640;
641, 641;
642, 642;
643, 643;
644, 644;
645, 645;
646, 646;
647, 647;
648, 648;
649, 649;
650, 650;
651, 651;
652, 652;
653, 653;
654, 654;
655, 655;
656, 656;
657, 657;
658, 658;
659, 659;
660, 660;
661, 661;
662, 662;
```

663, 663;
664, 664;
665, 665;
666, 666;
667, 667;
668, 668;
669, 669;
670, 670;
671, 671;
672, 672;
673, 673;
674, 674;
675, 675;
676, 676;
677, 677;
678, 678;
679, 679;
680, 680;
681, 681;
682, 682;
683, 683;
684, 684;
685, 685;
686, 686;
687, 687;
688, 688;
689, 689;
690, 690;
691, 691;
692, 692;
693, 693;
694, 694;
695, 695;
696, 696;
697, 697;
698, 698;
699, 699;
700, 700;
701, 701;
702, 702;
703, 703;
704, 704;
705, 705;
706, 706;
707, 707;
708, 708;
709, 709;
710, 710;
711, 711;
712, 712;
713, 713;
714, 714;
715, 715;

716, 716;
717, 717;
718, 718;
719, 719;
720, 720;
721, 721;
722, 722;
723, 723;
724, 724;
725, 725;
726, 726;
727, 727;
728, 728;
729, 729;
730, 730;
731, 731;
732, 732;
733, 733;
734, 734;
735, 735;
736, 736;
737, 737;
738, 738;
739, 739;
740, 740;
741, 741;
742, 742;
743, 743;
744, 744;
745, 745;
746, 746;
747, 747;
748, 748;
749, 749;
750, 750;
751, 751;
752, 752;
753, 753;
754, 754;
755, 755;
756, 756;
757, 757;
758, 758;
759, 759;
760, 760;
761, 761;
762, 762;
763, 763;
764, 764;
765, 765;
766, 766;
767, 767;
768, 768;

769, 769;
770, 770;
771, 771;
772, 772;
773, 773;
774, 774;
775, 775;
776, 776;
777, 777;
778, 778;
779, 779;
780, 780;
781, 781;
782, 782;
783, 783;
784, 784;
785, 785;
786, 786;
787, 787;
788, 788;
789, 789;
790, 790;
791, 791;
792, 792;
793, 793;
794, 794;
795, 795;
796, 796;
797, 797;
798, 798;
799, 799;
800, 800;
801, 801;
802, 802;
803, 803;
804, 804;
805, 805;
806, 806;
807, 807;
808, 808;
809, 809;
810, 810;
811, 811;
812, 812;
813, 813;
814, 814;
815, 815;
816, 816;
817, 817;
818, 818;
819, 819;
820, 820;
821, 821;

822, 822;
823, 823;
824, 824;
825, 825;
826, 826;
827, 827;
828, 828;
829, 829;
830, 830;
831, 831;
832, 832;
833, 833;
834, 834;
835, 835;
836, 836;
837, 837;
838, 838;
839, 839;
840, 840;
841, 841;
842, 842;
843, 843;
844, 844;
845, 845;
846, 846;
847, 847;
848, 848;
849, 849;
850, 850;
851, 851;
852, 852;
853, 853;
854, 854;
855, 855;
856, 856;
857, 857;
858, 858;
859, 859;
860, 860;
861, 861;
862, 862;
863, 863;
864, 864;
865, 865;
866, 866;
867, 867;
868, 868;
869, 869;
870, 870;
871, 871;
872, 872;
873, 873;
874, 874;

875, 875;
876, 876;
877, 877;
878, 878;
879, 879;
880, 880;
881, 881;
882, 882;
883, 883;
884, 884;
885, 885;
886, 886;
887, 887;
888, 888;
889, 889;
890, 890;
891, 891;
892, 892;
893, 893;
894, 894;
895, 895;
896, 896;
897, 897;
898, 898;
899, 899;
900, 900;
901, 901;
902, 902;
903, 903;
904, 904;
905, 905;
906, 906;
907, 907;
908, 908;
909, 909;
910, 910;
911, 911;
912, 912;
913, 913;
914, 914;
915, 915;
916, 916;
917, 917;
918, 918;
919, 919;
920, 920;
921, 921;
922, 922;
923, 923;
924, 924;
925, 925;
926, 926;
927, 927;

```
    928, 928;
    929, 929;
    930, 930;
    931, 931;
    932, 932;
    933, 933;
    934, 934;
    935, 935;
    936, 936;
    937, 937;
    938, 938;
    939, 939;
    940, 940;
    941, 941;
    942, 942;
    943, 943;
    944, 944;
    945, 945;
    946, 946;
    947, 947;
    948, 948;
    949, 949;
    950, 950;
    951, 951;
    952, 952;
    953, 953;
    954, 954;
    955, 955;
    956, 956;
    957, 957;
    958, 958;
    959, 959;
    960, 960;
    961, 961;
    962, 962;
    963, 963;
    964, 964;
    965, 965;
    966, 966;
    967, 967;
];

Zav_results = zeros(size(ranges, 1), numel(path));
I_results = zeros(size(ranges, 1), numel(path));

for i = 1:numel(path)
    data = readtable(path{i}, opts);
    data = table2array(data);

    MIC1 = data(1:x, 2);
    MIC2 = data(x+1:2*x, 2);
    MIC3 = data(2*x+1:3*x, 2);
    MIC4 = data(3*x+1:4*x, 2);
```

```
    MIC5 = data(4*x+1:5*x, 2);
    MIC6 = data(5*x+1:6*x, 2);
    MIC7 = data(6*x+1:7*x, 2);
    MIC8 = data(7*x+1:8*x, 2);
    MIC9 = data(8*x+1:9*x, 2);
    MIC10 = data(9*x+1:10*x, 2);
    MIC11 = data(10*x+1:11*x, 2);
    MIC12 = data(11*x+1:12*x, 2);
    MIC13 = data(12*x+1:13*x, 2);
    MIC14 = data(13*x+1:14*x, 2);
    MIC15 = data(14*x+1:15*x, 2);
    MIC16 = data(15*x+1:16*x, 2);
    MIC17 = data(16*x+1:17*x, 2);
    MIC18 = data(17*x+1:18*x, 2);

    for k = 1:size(ranges, 1)
        int = ranges(k, 1);
        fin = ranges(k, 2);

        I1 = mean(MIC1(int:fin));
        I2 = mean(MIC2(int:fin));
        I3 = mean(MIC3(int:fin));
        I4 = mean(MIC4(int:fin));
        I5 = mean(MIC5(int:fin));
        I6 = mean(MIC6(int:fin));
        I7 = mean(MIC7(int:fin));
        I8 = mean(MIC8(int:fin));
        I9 = mean(MIC9(int:fin));
        I10 = mean(MIC10(int:fin));
        I11 = mean(MIC11(int:fin));
        I12 = mean(MIC12(int:fin));
        I13 = mean(MIC13(int:fin));
        I14 = mean(MIC14(int:fin));
        I15 = mean(MIC15(int:fin));
        I16 = mean(MIC16(int:fin));
        I17 = mean(MIC17(int:fin));
        I18 = mean(MIC18(int:fin));

        Z_up = (6*I1) + (7*I2) + (8*I3) + (9*I4) + (10*I5) + (11*I6) +
               (12*I7) + (13*I8) + (14*I9) + (15*I10) + (16*I11) + (17*I12)
               + (18*I13) + (19*I14) + (20*I15) + (21*I16) + (22*I17) +
               (23*I18);

        Z_down = I1 + I2 + I3 + I4 + I5 + I6 + I7 + I8 + I9 + I10 + I11 +
                 I12 + I13 + I14 + I15 + I16 + I17 + I18;

        Z = Z_up ./ Z_down;

        Zav_results(k, i) = Z;
        I_results(k, i) = Z_down;
    end
end
```

```
for i = 1:numel(path)
    fprintf('Results for file %d:\n', i);
    fprintf('Zav results:\n');
    disp(Zav_results(:, i));
    fprintf('I results:\n');
    disp(I_results(:, i));
end
```
